# Outcomes of transcatheter vs surgical aortic valve replacement in pre-existing chronic liver disease patients: A meta-analysis of observational studies

**DOI:** 10.1016/j.ijcha.2025.101651

**Published:** 2025-03-28

**Authors:** Aizaz Ali, Muhammad Abdullah Ali, Asad Iqbal Khattak, Fazia Khattak, Abdullah Afridi, Touba Azeem, Umme Salma Shabbar Banatwala, Umama Alam, Ayesha Khan, Urbe Jalal, Abdul Moeez, Malik W.Z. Khan, Peter Collins, Raheel Ahmed

**Affiliations:** a Khyber Medical College, Peshawar, Pakistan; b Bacha Khan Medical College, Pakistan; c Dow University of Health Sciences, Pakistan; d Dow International Medical College, Pakistan; e Allama Iqbal Medical College, Pakistan; f National Heart and Lung Institute, Imperial College London, United Kingdom

**Keywords:** Chronic Liver Disease, Transcatheter Aortic Valve Replacement, Surgical Aortic Valve Replacement, Meta-analysis

## Abstract

Aortic valve stenosis in patients with chronic liver diseases, particularly liver cirrhosis and End-Stage Liver Disease, poses significant management challenges due to the interplay between cardiovascular and hepatic dysfunction. This systematic review and meta-analysis compared the safety and efficacy of Transcatheter Aortic Valve Replacement (TAVR) and Surgical Aortic Valve Replacement in this high-risk population. An extensive search of PubMed, Embase, and Web of Science (inception to January 5, 2025) identified 11 retrospective studies comprising 19,097 patients. Risk ratios for dichotomous outcomes and mean differences (MD) for continuous outcomes, each with 95% confidence intervals, were calculated using random-effects models.

The analysis revealed that TAVR significantly reduced hospital mortality (RR 0.36, 95 % CI: 0.30–0.42; I^2^ = 7.6 %), acute kidney injury (RR 0.51, 95 % CI: 0.33–0.78; I^2^ = 57.2 %), bleeding (RR 0.33, 95 % CI: 0.28–0.39; I^2^ = 0.0 %), stroke (RR 0.35, 95 % CI: 0.23–0.51; I^2^ = 6.1 %), and blood transfusion (RR 0.48, 95 % CI: 0.40–0.57; I^2^ = 7.6 %). TAVR was also associated with shorter hospital stays (MD −6.77 days, 95 % CI: −9.17 to −4.38; I^2^ = 97.5 %). No significant differences were observed in vascular complications requiring surgery or hospital charges and post-operative infections.

These findings suggest TAVR offers significant advantages over SAVR in reducing complications such as mortality, acute kidney injury, and bleeding in patients with liver disease. However, further randomized trials are necessary to confirm long-term outcomes and establish optimal treatment strategies for this high-risk population.

## 1 Introduction

Valvular heart diseases, particularly aortic valve stenosis, present unique challenges in patients with chronic liver diseases, especially liver cirrhosis and end-stage liver disease (ESLD), due to the complex interplay between cardiovascular and hepatic dysfunction [1]. Hepatic insufficiency, such as liver cirrhosis and ESLD, is responsible for complications like coagulopathy, bleeding episodes [2], symptomatic ascites, spontaneous bacterial peritonitis, hepatorenal syndrome [3], cerebral edema, and brain herniation [4]. Cirrhotic patients may also develop a hyperdynamic syndrome, characterized by an increased heart rate, elevated cardiac output, and decreased systemic vascular resistance and arterial blood pressure. Furthermore, cirrhosis can lead to “cirrhotic cardiomyopathy,” which impairs the contractile responsiveness of the cardiovascular system [5]. These complications pose major risks when patients with chronic hepatic diseases have to undergo AVR (Aortic Valve Replacement).

The traditional treatment modality for aortic valve replacement is Surgical Aortic Valve Replacement (SAVR). However, this option is highly invasive and poses several challenges, particularly for patients with hepatic insufficiency [5]. A more recent advancement, Transcatheter Aortic Valve Replacement (TAVR), mitigates many of the complications associated with SAVR and has shown promising results in high-risk patients with hepatic insufficiency [6]. TAVR is associated with a lower rate of permanent pacemaker implantation (PPI) [7], superior hemodynamic performance [8], and reduced major vascular complications [8]. In contrast, SAVR is linked to longer hospital stays, higher costs, increased rates of tracheotomy, and greater vasopressor use [9]. Although TAVR appears to be highly effective, there is a lack of solid evidence regarding its outcomes in this patient population.

The various differences between SAVR and TAVR have been analysed by different studies, comparing SAVR and TAVR [10], [11]. However, the applicability of the findings of these studies is severely reduced by their limitations. By pooling the data from various articles, this meta-analysis aims to determine whether TAVR is superior to SAVR in terms of mortality, perioperative outcomes, procedural complications, and adverse events. The findings of this analysis will be a cornerstone that guides future clinical practices and assists in identifying the optimal treatment strategies for patients with hepatic insufficiency, liver cirrhosis, ESLD, and chronic liver disease.

## 2 Methods

### 2.1 Study design and protocol registration

This systematic review followed the recommendations of the Cochrane Collaboration [12] and the Preferred Reporting Items for Systematic Reviews and Meta-Analysis (PRISMA) guidelines [13], including the design, implementation of the steps, analysis, and description of the results. The study protocol was registered in the International Prospective Register of Systematic Reviews (PROSPERO) under registration number CRD42025634863.

### 2.2 Search strategy and Databases

An electronic search of PubMed, Embase, and Web of Science, was conducted, covering all available entries from their inception to January 2024, without any language restrictions. The following keywords were used: ‘Chronic liver disease,’ ‘Hepatic insufficiency,’ ‘Liver diseases,’ ‘Liver cirrhosis,’ ‘Liver fibrosis,’ ‘Transcatheter aortic valve replacement,’ ‘TAVR,’ ‘TAVI,’ ‘Aortic Valve Stenosis,’ ‘Aortic Valve Insufficiency,’ and ‘Aortic Valve Incompetence.’ A comprehensive search strategy is available in the Supplementary file (Table S1).

### 2.3 Study selection and eligibility criteria

All studies obtained through the online search were imported into Rayyan software for screening, where duplicate entries were removed. The remaining articles underwent initial screening based on their titles and abstracts. Full-text articles were obtained if the abstracts were relevant to either investigator. Two reviewers (T.A. and A.K) independently evaluated each study's eligibility based on the inclusion criteria. Any discrepancies were resolved through discussion and agreement with a third researcher (A.A.). Eligible studies for this systematic review met the following criteria: (1) inclusion of patients with different stages of chronic liver diseases such as liver cirrhosis and ESLD; (2) interventions involving TAVR; (3) SAVR as a control; and (4) reporting at least one outcome of interest. Exclusion criteria were: (1) overlapping populations, defined by shared institutions and recruitment periods; (2) populations outside the scope of interest; (3) republished literature; (4) protocols without reported results; (5) reviews, abstracts, case reports, case series, background articles, expert opinions, or in vivo/in vitro studies; (6) duplicate data from the same clinical trial; or (7) absence of a comparator group.

### 2.4 Data Extraction and outcomes

Two authors (M.A. and T.A.) extracted data from the included studies into an Excel sheet using a pre-piloted form, which consisted of two sections: baseline data (first author name, year of publication, participants, intervention/control, mean age, disease stage, liver evaluation criteria, hypertension, diabetes, coronary artery disease (CAD), and atrial fibrillation). The primary outcomes of this study included in-hospital mortality, acute kidney injury (AKI), stroke, bleeding, and hospital length of stay. The secondary outcomes comprised blood transfusion, cardiac arrest, vascular complications requiring surgery, myocardial infarction (MI), hospital charges, cardiogenic shock, cardiac tamponade and post-operative infections.

### 2.5 Quality assessment

We assessed the risk of bias using the Newcastle-Ottawa Quality Assessment Form for observational Studies [14], which includes three domains: selection, comparability, and outcome. We applied thresholds to convert the Newcastle-Ottawa scores to AHRQ standards (good, fair, and poor). The assessment was independently performed by two reviewers (A.K. and U.J.), with disagreements resolved by discussion or consultation with a third reviewer (A.A.). The selection domain was rated with a maximum of four stars, the comparability domain with a maximum of two stars, and the outcome domain with a maximum of three stars. Studies scoring 7–9 stars were rated as “low risk of bias,” studies scoring 5–6 stars were rated as “some concerns,” and studies scoring less than 5 stars were rated as “high risk of bias,” ensuring a comprehensive evaluation [15].

### 2.6 Certainty of evidence

The Grading of Recommendations, Assessment, Development, and Evaluation (GRADE) tool was employed by two independent authors (A.I.K. and U.A.) using the GRADEpro Guideline Development Tool [16] to evaluate the level of certainty of the evidence in this meta-analysis, with categorizations ranging from high to very low [17]. Any disagreements were discussed and resolved through consensus.

### 2.7 Statistical analysis

Statistical analysis was performed using R software and R Studio (version 4.4.2, 2024.10.31 + 87279; R Core Team, Vienna, Austria), employing DerSimonian and Laird’s random-effects model to calculate pooled analyses with 95 % confidence intervals (CIs) [18]. The results were presented as pooled analyses in forest plots. Binary outcomes were assessed with risk ratios (RRs) and continuous outcomes with mean differences (MDs), with results displayed in forest plots. Heterogeneity was evaluated using the Cochrane Q chi-square test and the I2 statistic, with P-values < 0.10 and I2 > 50 % indicating significant heterogeneity [19]. We also used funnel plots to assess for publication bias.The stability of the pooled estimates was assessed through a leave-one-out analysis, in which data from each study were sequentially removed and the remaining dataset re-analyzed. This helped ensure that the aggregated effect sizes were not unduly influenced by any single study.

## 3 Results

### 3.1 Search results

**Fig. 1:**
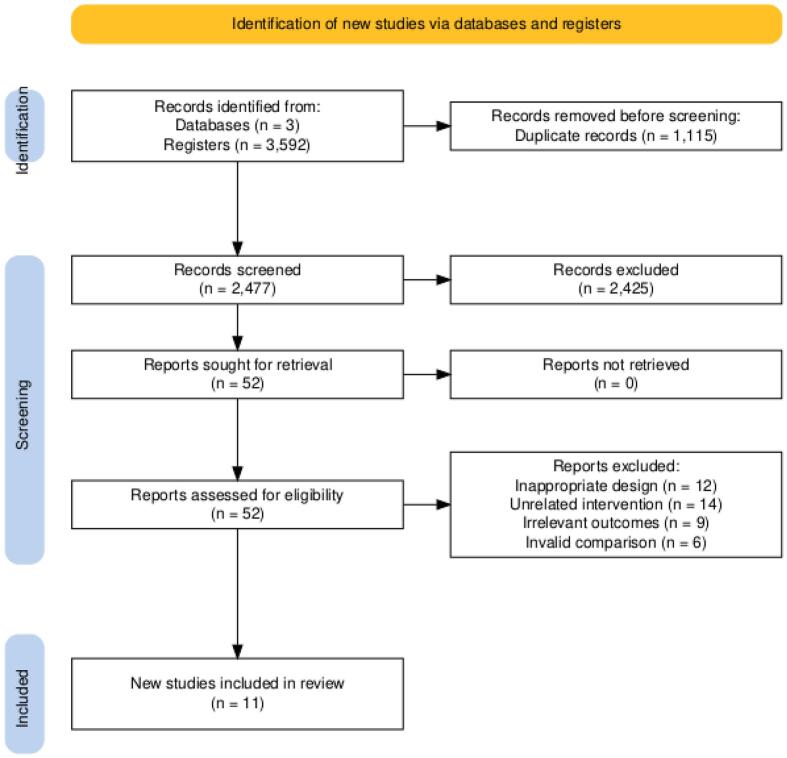
PRISMA flow chart.

The search identified 3,592 records. After removing 1,115 duplicate records, 2,477 articles were screened based on title and abstract. From this pool, 52 records were selected for full-text screening. Finally, eleven retrospective observational studies were included in the qualitative and quantitative synthesis [6], [10], [11], [12], [20], [21], [22], [23], [24], [25], [26], [27]. The study selection process is summarized in the PRISMA flow diagram (Fig. 1).

### 3.2 Baseline characteristics of included studies

The studies comprised a total of 19,097 patients, of whom 8,886 (46.53 %) underwent TAVR. The follow-up period extended up to X months. The mean age ranged from 62 to 75 years. The most common comorbidities were diabetes (7943.1, 41.59 %). The baseline characteristics of the included studies are detailed in Table 1.

**Table 1:** Baseline characteristics of included studies.

**Study id**	**Country**	**Study design**	**Sample size, n**		**Mean age in years (S.D)**		**Disease type**	**Time course**	**Liver evaluation criteria**	**Diabetes mellitus, n (%)**		**Hypertension, n (%)**		**CAD, n (%)**		**Atrial fibrillation, n (%)**		**PVD, n (%)**	
			**TAVR**	**SAVR**	**TAVR**	**SAVR**				**TAVR**	**SAVR**	**TAVR**	**SAVR**	**TAVR**	**SAVR**	**TAVR**	**SAVR**	**TAVR**	**SAVR**
Aggarwal 2024	USA	Cohort (propensity)	359	359	67.3(7.8)	66.2(8.8)	Liver cirrhosis	2016–19	ICD-10-CM, ICD-10-PCS	157(43.7)	152(42.3)	171(47.6)	169(47.1)	144(40.1)	131(36.5)	163(45.4)	169(47.1)	NR	NR
Alhaqtani 2017	USA	Cohort (propensity)	134	134	72(10)	64(13)	Liver cirrhosis	2003 to 2014	ICD-9-CM	88(50.6)	447(28.1)	124(71.3)	785(49.3)	1(0.6)	3(0.2)	63(36.2)	659(41.4)	NR	NR
Ali 2024	USA	Cohort (propensity)	7,109	7,655	70.33(9.64)	65.6(11.86)	Liver cirrhosis	2016 to 2020	ICD-10 CM	3,490 (49.1)	2,597 (33.9)	4,825 (67.9)	3,493 (45.6)	33.9 (61.8)	23.9 (43.6)	NR	NR	NR	NR
Dhoble 2017	USA	Cohort (propensity)	55	55	67.2*	67*	Liver cirrhosis	2012 to 2014	ICD-9-CM	32 (58.2)	30 (54.5)	NR	NR	NR	NR	NR	NR	NR	NR
Greason 2013	USA	Cohort	6	12	71.00 (20.02)	70.33(19.29)	Liver cirrhosis	2008 to 2012	CTP and MELD	3 (50)	3 (25)	NR	NR	NR	NR	NR	NR	4 (67)	2 (17)
Khan 2020	USA	Cohort (propensity)	273	404	67.5(8.6)	65.4(9.2)	ESLD	2005 to 2015	ICD-9-CM	<11(<4)	<11(<2.7)	175(63.9)	230(56.9)	NR	NR	NR	NR	45(16.5)	59(14.6)
Lee 2021	USA	Cross-sectional	606	1353	72.5*	62.8*	CLD	2011 to 2017	ICD-9-CM and ICD-10-CM	316.9 (52.3)	492.2 (36.4)	218.16 (36)	660 (48.8)	359.9 (59.4)	671 (49.6)	NR	NR	NR	NR
Peeraphatdit 2020	USA	Cohort (propensity)	55	50	75.4 (9.4)	68.4 (8.7)	Liver cirrhosis	2008–2016	MELD	31 (56.4)	20 (40.0)	47 (85.5)	35 (70.0)	31 (56.4)	13 (26.0)	NR	NR	28 (50.9)	2 (4.0)
Seppelt 2019	Germany	Cohort	43	42	74.3 (10.67)	71.6 (7.9)	preexisting liver disease (acute or chronic hepatitis, fatty liver, liver cirrhosis)	2004–2016	Child-Pugh, MELD	18 (40.4)	12 (28.6)	NR	NR	25 (55.8)	16 (38.1)	18 (41.9)	8 (19.1)	8 (18.6)	3 (7.1)
Thakkar 2015	USA	Cross-sectional (propensity)	30	30	71.73 (10.02)	70.47 (6.19)	Liver cirrhosis	2011–2012	ICD-9-CM	16 (53.3)	16 (53.3)	20 (66.7)	22 (73.3)	44(90)	5(100)	NR	NR	6 (20)	3 (10)
Winte 2020	USA	Cohort	216	117	69.5 (11.8)	65.9 (11.9)	Liver cirrhosis	2012–2017	APRI, FIB-4	NR	NR	49(100)	5(100)	NR	NR	23(47)	2(40)	9(18)	1(20)

ICD-10-CM = International Classification of Diseases, 10th Revision, Clinical Modification

ICD-9-CM = International Classification of Diseases, 9th Revision, Clinical Modification

ICD-10-PCS = International Classification of Diseases, 10th Revision, Procedure Coding System.

MELD = Model for End-Stage Liver Disease.

APRI = Aspartate Aminotransferase to Platelet Ratio Index.

FIB = Fibrosis-4 Index.

CTP=Child-Turcotte-Pugh Score.

ESLD = End stage liver disease.

CLD=Chronic liver disease.

CAD=Coronary artery disease.

PVD=Previous vascular disease.

* Mean without S.D.

### 3.3 Meta-analysis

#### 3.3.1 In-hospital mortality

**Fig. 2:**
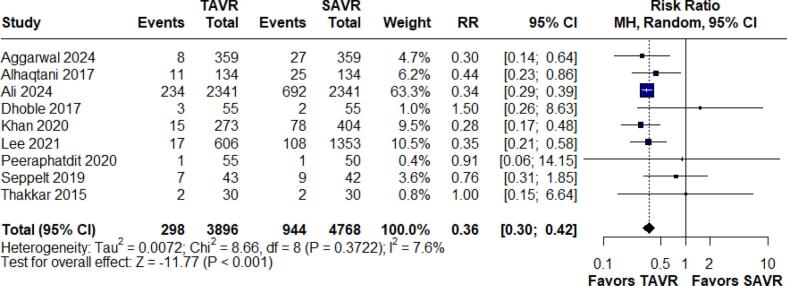
Forest Plot for In-hospital Mortality.

Hospital mortality was assessed in nine studies comprising 8,664 patients. The pooled results demonstrated that TAVR significantly reduces the risk of hospital mortality compared to SAVR, with a risk ratio (RR) of 0.36 (95 % CI: 0.30–0.42; p < 0.001, I^2^ = 7.6 %). (Fig. 2).

#### 3.3.2 Acute kidney injury

**Fig. 3:**
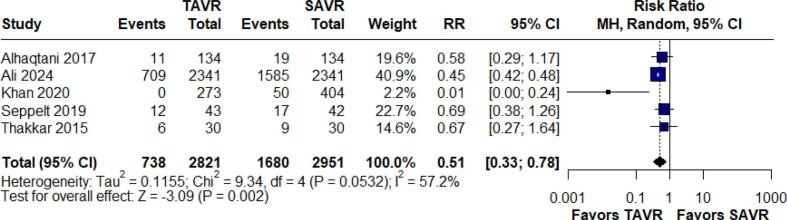
Forest Plot for Acute Kidney Injury.

Acute kidney injury (AKI) was evaluated in five studies involving 5,772 patients. The analysis revealed that TAVR is associated with a lower rate of AKI relative to SAVR, with an RR of 0.51 (95 % CI: 0.33–0.78; p < 0.002, I^2^ = 57.2 %) (Fig. 3). Given that high heterogeneity, leave-one-out analyses were performed and heterogeneity dropped to low (I^2^ = 5.1 %) (Supplementary Fig. S12).

#### 3.3.3 Blood transfusion

**Fig. 4:**
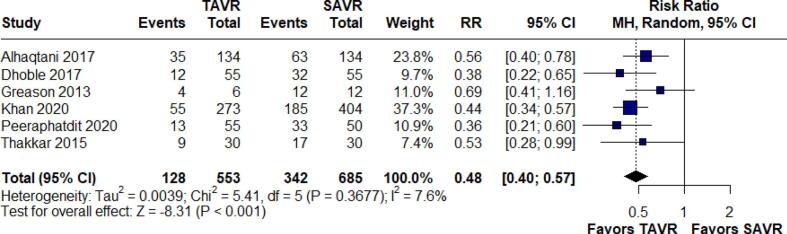
Forest Plot for Blood Transfusion.

Blood transfusion outcomes were reported in six studies consisting of 1,238 patients. The pooled analysis indicated a significant reduction in the risk of blood transfusion with TAVR compared to SAVR, with an RR of 0.48 (95 % CI: 0.40–0.57; p < 0.001, I^2^ = 7.6 %). (Fig. 4).

#### 3.3.4 Stroke

**Fig. 5:**
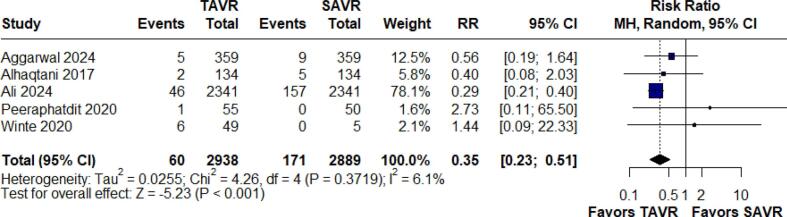
Forest Plot for Stroke.

Stroke outcomes were analyzed in five studies with a total of 5,827 patients. The findings showed that TAVR significantly reduces the risk of stroke relative to SAVR, with an RR of 0.35 (95 % CI: 0.23–0.51; p < 0.001, I^2^ = 6.1 %). (Fig. 5).

#### 3.3.5 Cardiac arrest

**Fig. 6:**
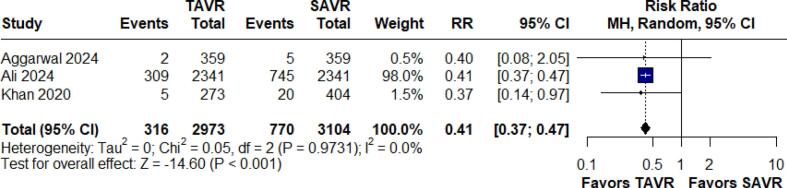
Forest Plot for Cardiac Arrest.

Cardiac arrest was examined in three studies involving 6,077 patients. The pooled results demonstrated that TAVR is associated with a lower risk of cardiac arrest compared to SAVR, with an RR of 0.41 (95 % CI: 0.37–0.47; p < 0.001, I^2^ = 0.0 %). (Fig. 6).

#### 3.3.6 Bleeding

**Fig. 7:**
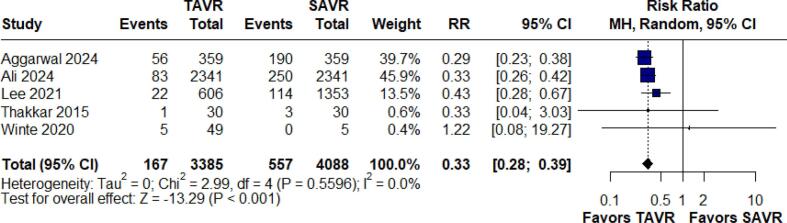
Forest Plot for Bleeding.

Bleeding outcomes were assessed in five studies comprising 7,477 patients. The analysis showed that TAVR significantly reduces the risk of bleeding relative to SAVR, with an RR of 0.33 (95 % CI: 0.28–0.39; p < 0.001, I^2^ = 0.0 %). (Fig. 7).

Post-Operative Infections

**Fig. 8:**
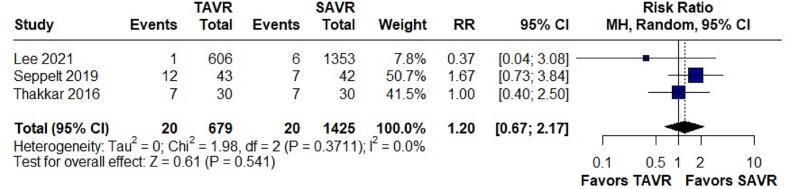
Forest Plot for Post Operative Infections.

Postoperative infections were assessed in three studies comprising 2,104 patients. The analysis showed no significant difference in the risk of postoperative infections between TAVR and SAVR, with an RR of 1.20 (95 % CI: 0.67–2.17; p = 0.541, I^2^ = 0.0 %) (Fig. 8).

#### 3.3.7 Additional Outcomes

A pooled analysis demonstrated that the risk of vascular complications was lower with TAVR relative to SAVR (RR 0.54; 95 % CI 0.30–––0.98; p < 0.044; I^2^ = 46.7 %); as well as a shorter hospital stay (MD −6.77 days; 95 % CI −9.17 to −4.38; p < 0.001; I^2^ = 97.5 %). Conversely, a greater number of pacemaker implantations were observed in the SAVR group (RR 1.33; 95 % CI 0.79–––2.24; p = 0.29; I^2^ = 59.3 %; [Fig. 2A]). However, no significant difference was observed in vascular complications requiring surgery (RR 1.17; 95 % CI 0.41–3.40; p = 0.767; I^2^ = 0.0 %), myocardial infarction (RR 0.68; 95 % CI 0.32–1.45; p = 0.321; I^2^ = 63.1 %), hospital charges (MD −58,277.64 dollars; 95 % CI −127,341.21 to 10,785.92; p = 0.10; I^2^ = 99.7 %), cardiogenic shock (RR 0.63; 95 % CI 0.32–1.23; p = 0.175; I^2^ = 78.3 %), or cardiac tamponade (RR 0.91; 95 % CI 0.37–2.25; p = 0.847; I^2^ = 0.0 %). The forest plots of these additional outcomes are available in the supplementary file (Figs. S1 to S8).

#### 3.3.8 Quality Assessment

The risk of bias for each included study was assessed using the Newcastle-Ottawa Scale, and the majority of studies demonstrated a low risk of bias across all domains. However, *Seppelt et al. (2019)* exhibited limitations, particularly in the comparability domain. Despite these concerns, the study provided valuable data, and its inclusion in the meta-analysis highlights the need for cautious interpretation of its findings. Overall, the remaining studies had a low risk of bias, supporting the robustness of the meta-analysis. A detailed assessment of all the included studies is provided in the Supplementary File (Table S3).

#### 3.3.9 Certainty of evidence and publication Bias

The GRADE approach, using the GRADEpro Guideline Development Tool, was employed to assess the certainty of evidence. A detailed assessment is shown in Supplementary Table 2. For publication bias, we used funnel plots given in the supplementary files (F1-F14).

#### 3.3.10 Sensitivity Analysis

We performed a leave-one-out sensitivity analysis to assess the influence of individual studies on the pooled results. The removal of any single study had minimal impact on most outcomes. Excluding outliers significantly reduced heterogeneity in the outcomes of cardiogenic shock, myocardial infarction, and number of pacemaker implantations. Details of the sensitivity analyses for these three outcomes are provided in the Supplementary file (Figs. S9-S11).

## 4 Discussion

This systematic review and meta-analysis provides valuable insights into the efficacy and safety of TAVR compared to SAVR for the management of aortic valve abnormalities in patients with chronic liver disease. The findings reveal that TAVR is associated with a lower risk of acute kidney injury, cardiac arrest, in-hospital mortality, stroke, and vascular complications. Additionally, TAVR is linked to reduced blood transfusion requirements, less bleeding, and shorter hospital stays. Notably, the study also highlights comparable rates of vascular complications requiring surgery, myocardial infarction, hospital charges, cardiogenic shock, and cardiac tamponade between TAVR and SAVR.

A couple of similar reviews have compared TAVR and SAVR offering useful points for comparison. For instance, Jiang et al. [28] examined the impact of hepatic insufficiency on prognosis after TAVR and concluded that TAVR resulted in a lower risk of mortality, fewer blood transfusions, and reduced incidence of AKI. Ndunda et al. [29] reported consistent findings, noting a lower rate of in-hospital mortality, decreased need for blood transfusions, and shorter hospital stays with TAVR. Their study also found comparable rates of AKI, hospitalization costs, and discharge rates between TAVR and SAVR. Similarly, Ma et al. [30] observed lower in-hospital mortality and reduced transfusion requirements with TAVR. However, their analysis differed from ours, reporting similar rates of AKI, neurological complications, and vascular complications in both groups. It is important to note that these previous reviews included fewer studies than ours, which may have impacted the overall credibility and generalizability of their findings.

The reduced risk of AKI and bleeding complications associated with TAVR is especially pertinent in chronic liver disease patients who often have baseline coagulopathy and impaired renal function [23]. TAVR’s minimally invasive approach likely reduces the physiological stress and inflammatory response seen in open-heart surgeries, thereby lowering the risk of perioperative complications [31]. Additionally, shorter hospital stays following TAVR may help reduce the likelihood of hospital-acquired complications, such as infections or further liver decompensation, which are significant concerns in patients with liver disease [32].

Open-heart surgery, including SAVR, is inherently more disruptive to cardiovascular physiology compared to the percutaneous nature of TAVR. During open-heart surgery, hemodynamic function is maintained using non-pulsatile, low-flow circulation provided by the extracorporeal circulation system. In contrast, TAVR does not require extracorporeal circulation, reducing the risks of microvascular dysfunction, hemodynamic compromise, and subsequent inflammation and myocardial injury. Extracorporeal circulation during SAVR can lead to decreased oxygen saturation in the venule microvasculature, promoting ischemia and inflammation, which increases the risk of cardiovascular events such as cardiogenic shock, sudden cardiac arrest, and hemodynamic instability [33]. Furthermore, while SAVR requires general anesthesia and cardiopulmonary bypass, TAVR is increasingly performed under local anesthesia with conscious sedation, offering substantial benefits in chronic liver disease patients, who are at heightened risk for anesthesia-related complications, including postoperative respiratory failure and cardiac issues. [34], [35] Although our study showed a higher risk of cardiac arrest with SAVR, the risk of cardiogenic shock and MI was comparable between both groups. Despite TAVR’s less invasive nature, the risk of MI remains due to procedural complications, such as coronary obstruction or embolization [36]. Both procedures involve patients with significant baseline coronary artery disease, which likely contributes to the similar rates of MI observed between TAVR and SAVR. These findings suggest that while TAVR is associated with fewer systemic complications, certain procedural risks remain, which must be carefully considered when choosing the most appropriate intervention for individual patients.

The similar rates of vascular complications requiring surgical intervention between TAVR and SAVR highlight the critical need for precise technical execution during TAVR to avoid device-related complications. Additionally, the ongoing risks of cardiogenic shock and cardiac tamponade in both procedures emphasize the necessity of careful perioperative management in these high-risk patients. Choosing less invasive devices, such as passive temporary pacemaker wires instead of screw-in leads, and optimizing the use of stiff wires can help minimize trauma during TAVR. Furthermore, pre-procedural assessments should thoroughly evaluate patient-specific factors such as annular size, calcification, left ventricular hypertrophy, and myocardial ischemia to ensure the best possible outcomes. Operator experience is also vital, as the learning curve associated with TAVR can significantly impact the rate of complications [37].

The relative risk (RR) of 1.20 (95 % CI: 0.67–2.17; p = 0.541, I^2^ = 0.0 %) suggests that there is no statistically significant difference in the risk of postoperative infections between TAVR and SAVR in patients with chronic liver disease. This is consistent with studies showing that neither TAVR nor SAVR appears to offer a clear advantage in terms of reducing postoperative infections in this patient population [38]. The impact of TAVI versus SAVR on the incidence of perioperative infections in cirrhotic patients is an important consideration, particularly due to the potential for microbiota-induced intermittent bacteremia affecting prosthetic valves. However, due to a lack of adequate data, we were unable to analyze this outcome in our study. Nevertheless, it is important to recognize that cirrhotic patients are inherently prone to bloodstream infections, with enteric Gram-negative bacteria being a common cause due to intestinal bacterial translocation [39]. This vulnerability could potentially affect prosthetic valves, irrespective of the implantation method. Additionally, the altered gut microbiome in cirrhotic patients—specifically, the reduction in short-chain fatty acid (SCFA)-producing bacteria—may contribute to increased intestinal permeability and bacterial translocation [40]. This, in turn, could theoretically elevate the risk of intermittent bacteremia and subsequent prosthetic valve infections.

This review boasts several strengths. First, the inclusion of a larger number of recent studies enhances the credibility of the findings compared to previous reviews. Second, the broad range of outcomes assessed provides clinicians with a comprehensive understanding of the two procedures’ advantages and limitations. Third, sensitivity analyses were conducted to ensure the robustness of our results. Furthermore, the majority of included studies were assessed as having a low risk of bias, minimizing confounding factors and improving the reliability of the conclusions. However, this review is not without limitations. Many of the studies included in the analysis were retrospective, which could introduce biases related to patient selection and reporting. Moreover, some studies drew from the same database with overlapping time frames, which could introduce duplicity into the analysis. Additionally, the lack of granular data on liver disease severity (e.g., MELD or Child-Pugh scores) limits our ability to draw conclusions for subpopulations with varying degrees of liver dysfunction. Furthermore, long-term outcomes such as valve durability, survival and quality of life were not reported consistently, thus restricting our ability to comprehend and study the long-lasting benefits associated with TAVR and SAVR. Future studies conducted on this topic should address these limitations by including detailed liver severity assessments and following-up on the patients for reporting long-term outcomes.

## 5 Conclusion

This systematic review and meta-analysis comparing TAVR and SAVR in patients with chronic liver disease presents us with compelling evidence that favors TAVR as a safer and more efficacious treatment option. Extensive analysis of data from the 11 included observational studies, involving 19,000 patients, indicates that TAVR significantly reduces hospital mortality, acute kidney injury, and other complications compared to SAVR, with risk ratios demonstrating substantial benefits across multiple outcomes. These results not only bring to light the potential TAVR holds in improving patient safety and recovery times but also underscore the necessity for further randomized controlled trials to establish long-term outcomes and refine treatment strategies for this high-risk population, which would further establish exemplary clinical practices in managing aortic valve disease amidst hepatic dysfunction.

## Clinical trial Registration

Not Applicable.

## Ethics Approval and Consent to Participate

Not Applicable.

## Data Availability Statement

All relevant data, including individual study characteristics, sample sizes, and effect sizes, are included within the article and its supplementary materials.

## CRediT authorship contribution statement

**Aizaz Ali:** Writing – review & editing, Writing – original draft, Software, Resources, Project administration, Methodology, Investigation, Formal analysis, Conceptualization. **Muhammad Abdullah Ali:** Writing – review & editing, Writing – original draft, Software, Resources, Project administration, Methodology, Formal analysis. **Asad Iqbal Khattak:** Writing – original draft, Software, Resources, Project administration, Methodology, Investigation, Formal analysis. **Fazia Khattak:** Writing – review & editing, Writing – original draft, Project administration, Methodology, Investigation, Formal analysis. **Abdullah Afridi:** Writing – original draft, Project administration, Methodology, Investigation, Formal analysis, Data curation. **Touba Azeem:** Writing – review & editing, Writing – original draft, Software, Resources, Methodology, Formal analysis, Data curation. **Umme Salma Shabbar Banatwala:** Writing – original draft, Resources, Project administration, Methodology, Investigation, Formal analysis. **Umama Alam:** Writing – original draft, Software, Resources, Methodology, Investigation. **Ayesha Khan:** Writing – review & editing, Writing – original draft, Resources, Methodology, Investigation, Formal analysis. **Urbe Jalal:** Writing – review & editing, Writing – original draft, Software, Resources, Formal analysis, Data curation. **Abdul Moeez:** Writing – review & editing, Writing – original draft, Software, Resources, Methodology. **Malik W.Z. Khan:** Writing – review & editing, Writing – original draft, Software, Resources, Methodology. **Peter Collins:** Writing – review & editing, Writing – original draft, Software, Resources. **Raheel Ahmed:** Writing – review & editing, Software, Resources, Methodology.

## Funding

This work received no funding.

## Declaration of competing interest

The authors declare that they have no known competing financial interests or personal relationships that could have appeared to influence the work reported in this paper.
