# Supplementary material for: Outcomes of transcatheter vs surgical aortic valve replacement in pre-existing chronic liver disease patients: A meta-analysis of observational studies
Source: Int J Cardiol Heart Vasc. 2025 Mar 28;58:101651. doi: 10.1016/j.ijcha.2025.101651 (PMC11994331; doi:10.1016/j.ijcha.2025.101651)
Supplement: Supplementary Data 1 [file mmc1.docx]

**Supplementary File:**

**Outcomes of TAVR vs SAVR in pre-existing chronic liver disease patients: A meta-analysis of observational studies.**

**Table S1** Detailed search strategy

| **Database** | **Search** | **Items Found** |
| --- | --- | --- |
| Pubmed | ((((Hepatic insufficiency [Mesh]) OR (Hepatic insufficiency OR Liver insufficiency [Title/Abstract])) OR ((Liver diseases [Mesh]) OR (Liver diseases OR Liver disease OR Liver dysfunction OR Liver dysfunctions [Title/Abstract]))) OR ((Liver cirrhosis [Mesh]) OR (Hepatic cirrhosis OR Liver fibrosis [Title/Abstract]))) AND (((((Transcatheter aortic valve replacement [Mesh]) OR (Transcatheter aortic valve replacement OR Transcatheter aortic valve implantation OR TAVR OR TAVI [Title/Abstract])) OR ((Aortic Valve Stenosis [Mesh]) OR (Aortic Valve Stenosis OR Aortic Valve Stenoses OR Aortic Stenosis))) OR ((Aortic Valve Insufficiency [Mesh]) OR (Aortic Valve Incompetence OR Aortic Incompetence OR Aortic Regurgitation OR Aortic Valve Insufficiency))) OR ((Aortic Valve [Mesh]) OR (Aortic Valves OR Aortic Valve [Title/Abstract]))) | 925 |
| Embase | ('hepatic insufficiency'/exp OR 'hepatic insufficiency':ti,ab OR 'liver insufficiency':ti,ab OR 'liver disease'/exp OR 'liver diseases':ti,ab OR 'liver disease':ti,ab OR 'liver dysfunction':ti,ab OR 'liver dysfunctions':ti,ab OR 'liver cirrhosis'/exp OR 'hepatic cirrhosis':ti,ab OR 'liver fibrosis':ti,ab) AND ('transcatheter aortic valve implantation'/exp OR 'transcatheter aortic valve replacement':ti,ab OR 'transcatheter aortic valve implantation':ti,ab OR 'tavr':ti,ab OR 'tavi':ti,ab OR 'aortic valve stenosis'/exp OR 'aortic valve stenosis':ti,ab OR 'aortic valve stenoses':ti,ab OR 'aortic stenosis':ti,ab OR 'aortic valve insufficiency'/exp OR 'aortic valve incompetence':ti,ab OR 'aortic incompetence':ti,ab OR 'aortic regurgitation':ti,ab OR 'aortic valve insufficiency':ti,ab OR 'aortic valve'/exp OR 'aortic valves':ti,ab OR 'aortic valve':ti,ab) | 2413 |
| Web of Science | TS=((("Hepatic insufficiency") OR ("Liver insufficiency") OR ("Liver diseases") OR ("Liver disease") OR ("Liver dysfunction") OR ("Liver dysfunctions") OR ("Liver cirrhosis") OR ("Hepatic cirrhosis") OR ("Liver fibrosis")) AND (("Transcatheter aortic valve replacement") OR ("Transcatheter aortic valve implantation") OR ("TAVR") OR ("TAVI") OR ("Aortic Valve Stenosis") OR ("Aortic Valve Stenoses") OR ("Aortic Stenosis") OR ("Aortic Valve Insufficiency") OR ("Aortic Valve Incompetence") OR ("Aortic Incompetence") OR ("Aortic Regurgitation") OR ("Aortic Valves") OR ("Aortic Valve"))) | 254 |

**Table S2** Detailed Assessment of our findings

| **Summary of findings:** | | | | | | |
| --- | --- | --- | --- | --- | --- | --- |
| **TAVR compared to SAVR for Patients with pre-existing Chronic liver disease** | | | | | | |
| **Patient or population:** Patients with pre-existing chronic liver disease  **Setting:**  **Intervention:** TAVR  **Comparison:** SAVR | | | | | | |
| Outcomes | **Anticipated absolute effects^*^** (95% CI) | | Relative effect  (95% CI) | № of participants  (studies) | Certainty of the evidence  (GRADE) | Comments |
|  | **Risk with SAVR** | **Risk with TAVR** |  |  |  |  |
| Acute kidney injury  assessed with: RR | 569 per 1,000 | **290 per 1,000**  (188 to 444) | **RR 0.51**  (0.33 to 0.78) | 5772  (5 non-randomized studies) | ⨁⨁⨁◯  Moderate |  |
| Blood transfusion  assessed with: RR | 499 per 1,000 | **1000 per 1,000**  (200 to 285) | **RR 48.00**  (0.40 to 0.57) | 1238  (6 non-randomized studies) | ⨁⨁⨁◯  Moderate |  |
| Bleeding  assessed with: RR | 136 per 1,000 | **45 per 1,000**  (38 to 53) | **RR 0.33**  (0.28 to 0.39) | 7473  (5 non-randomized studies) | ⨁⨁⨁◯  Moderate |  |
| Cardiac arrest  assessed with: RR | 248 per 1,000 | **102 per 1,000**  (92 to 117) | **RR 0.41**  (0.37 to 0.47) | 6077  (3 non-randomized studies) | ⨁⨁⨁◯  Moderate |  |
| Cardiogenic shock  assessed with: RR | 328 per 1,000 | **206 per 1,000**  (105 to 403) | **RR 0.63**  (0.32 to 1.23) | 6137  (4 non-randomized studies) | ⨁⨁◯◯  Low^a^ |  |
| Hospital mortality  assessed with: RR | 198 per 1,000 | **71 per 1,000**  (59 to 83) | **RR 0.36**  (0.30 to 0.42) | 8664  (9 non-randomised studies) | ⨁⨁⨁◯  Moderate |  |
| Stroke  assessed with: RR | 59 per 1,000 | **21 per 1,000**  (14 to 30) | **RR 0.35**  (0.23 to 0.51) | 5827  (5 non-randomised studies) | ⨁⨁⨁◯  Moderate |  |
| ***The risk in the intervention group** (and its 95% confidence interval) is based on the assumed risk in the comparison group and the **relative effect** of the intervention (and its 95% CI).   **CI:** confidence interval; **MD:** mean difference; **RR:** risk ratio | | | | | | |
| **GRADE Working Group grades of evidence**  **High certainty:** we are very confident that the true effect lies close to that of the estimate of the effect.  **Moderate certainty:** we are moderately confident in the effect estimate: the true effect is likely to be close to the estimate of the effect, but there is a possibility that it is substantially different.  **Low certainty:** our confidence in the effect estimate is limited: the true effect may be substantially different from the estimate of the effect.  **Very low certainty:** we have very little confidence in the effect estimate: the true effect is likely to be substantially different from the estimate of effect. | | | | | | |

**Table S3:** The risk of bias assessment of each included study

| **STUDY ID** | **SELECTION** | | | | **COMPATIBILITY** | | **OUTCOMES** | | | **TOTAL** | **OVERALL RISK** |
| --- | --- | --- | --- | --- | --- | --- | --- | --- | --- | --- | --- |
|  | **S1** | **S2** | **S3** | **S4** | **C1** | **C2** | **O1** | **O2** | **O3** |  |  |
| Dhoble 2017 | * | * | * | * | * | - | * | * | * | 8 | Low risk |
| Greason 2013 | * | * | * | * | * | - | * | * | * | 8 | Low risk |
| Khan 2020 | * | * | * | * | * | * | * | * | * | 9 | Low risk |
| Lee 2021 | * | * | * | * | * | * | * | * | * | 9 | Low risk |
| Aggarwal 2024 | * | * | * | * | * | * | * | * | * | 9 | Low risk |
| Alhaqtani 2017 | * | * | * | * | * | - | * | * | * | 8 | Low risk |
| Thakkar 2015 | - | * | * | * | * | * | * | * | * | 8 | Low risk |
| Winte 2020 | * | * | * | * | - | - | * | * | * | 7 | Low risk |
| Peeraphatdit 2020 | * | * | * | * | * | * | * | * | * | 9 | Low risk |
| Seppelt 2019 | * | * | * | - | * | - | * | * | - | 6 | Some concerns |
| Ali 2024 | * | * | * | - | * | * | * | * | - | 7 | Low risk |

**Funnel Plots:**

**Figure F1:** Funnel Plot for vascular complications requiring surgery

**
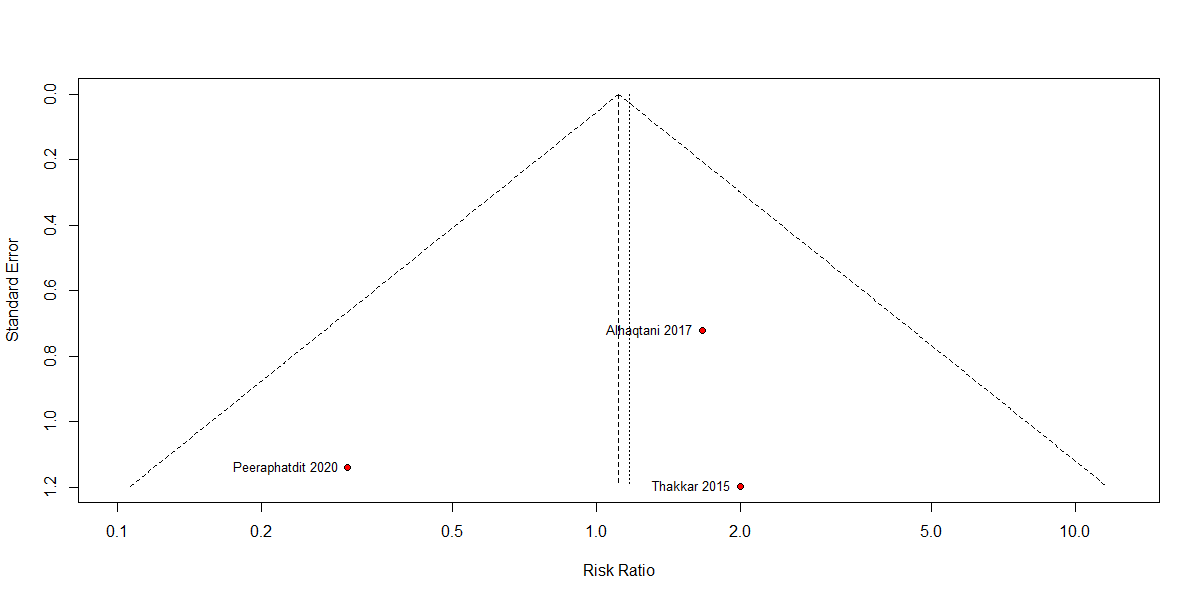
**

**Figure F2:** Funnel Plot for vascular complications


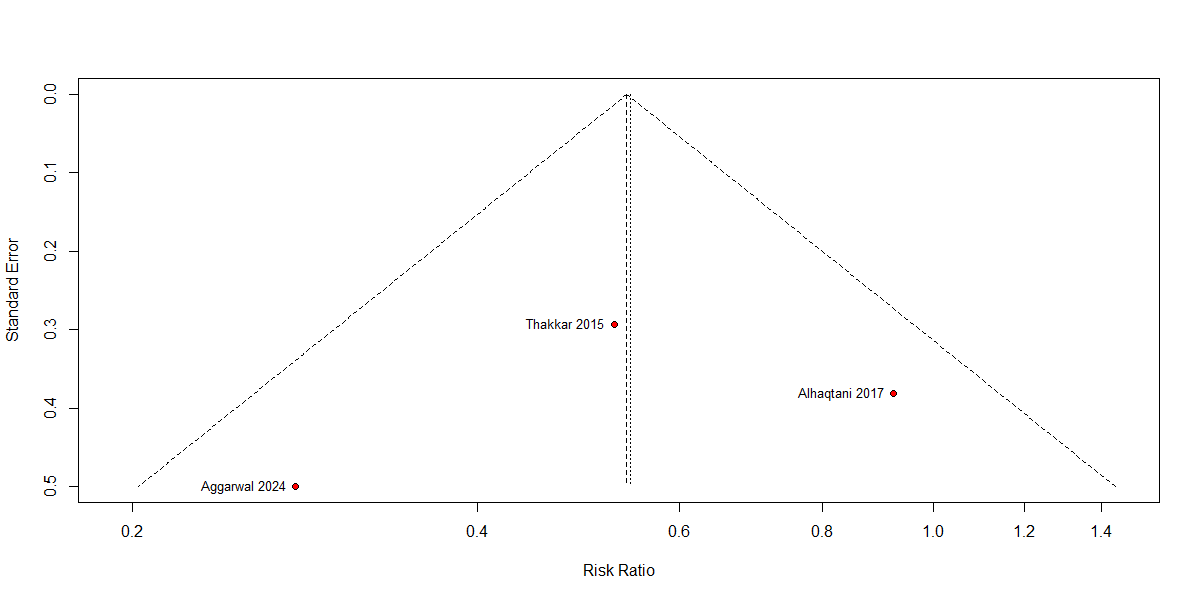


**Figure F3:** Funnel Plot for Stroke


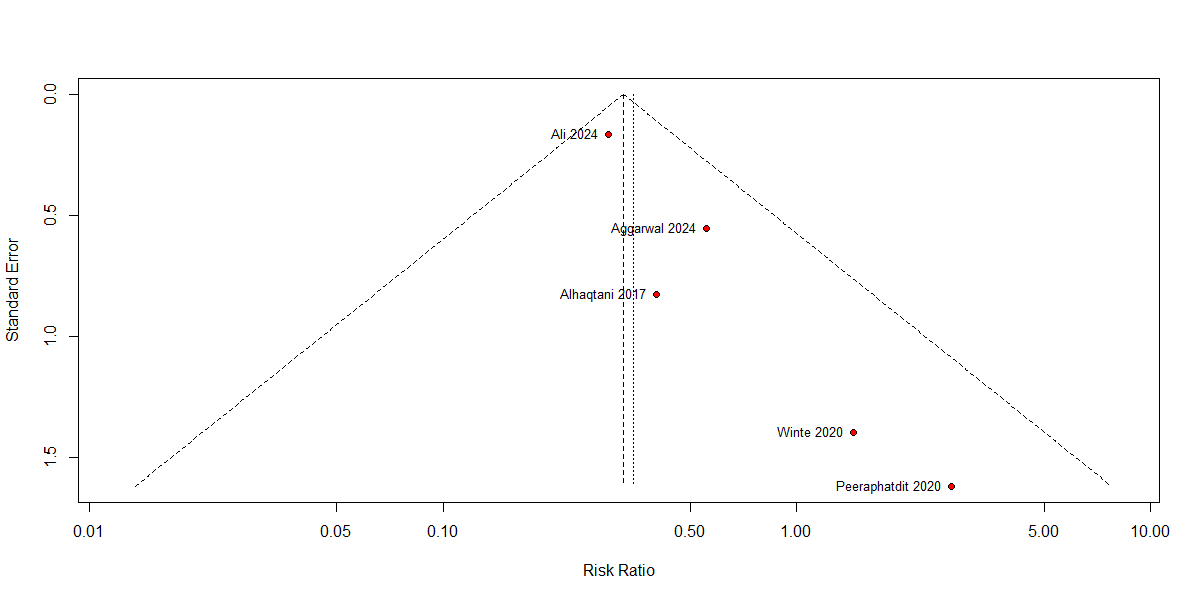


**Figure F4:** Funnel Plot for Stroke Procedural Death


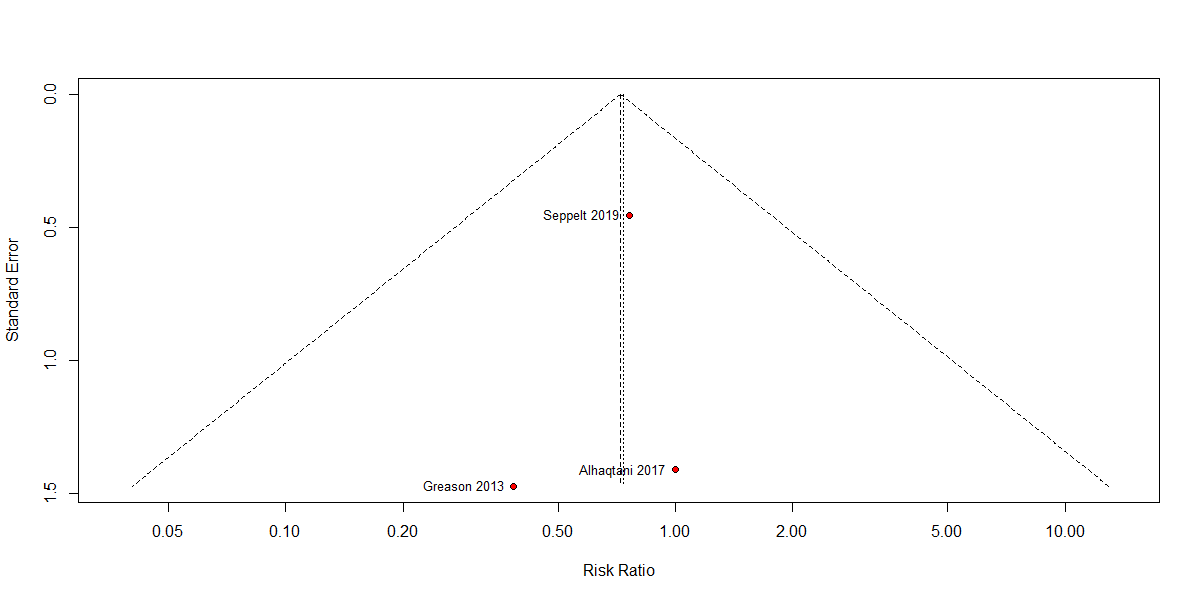


**Figure F5:** Funnel Plot for Pacemaker


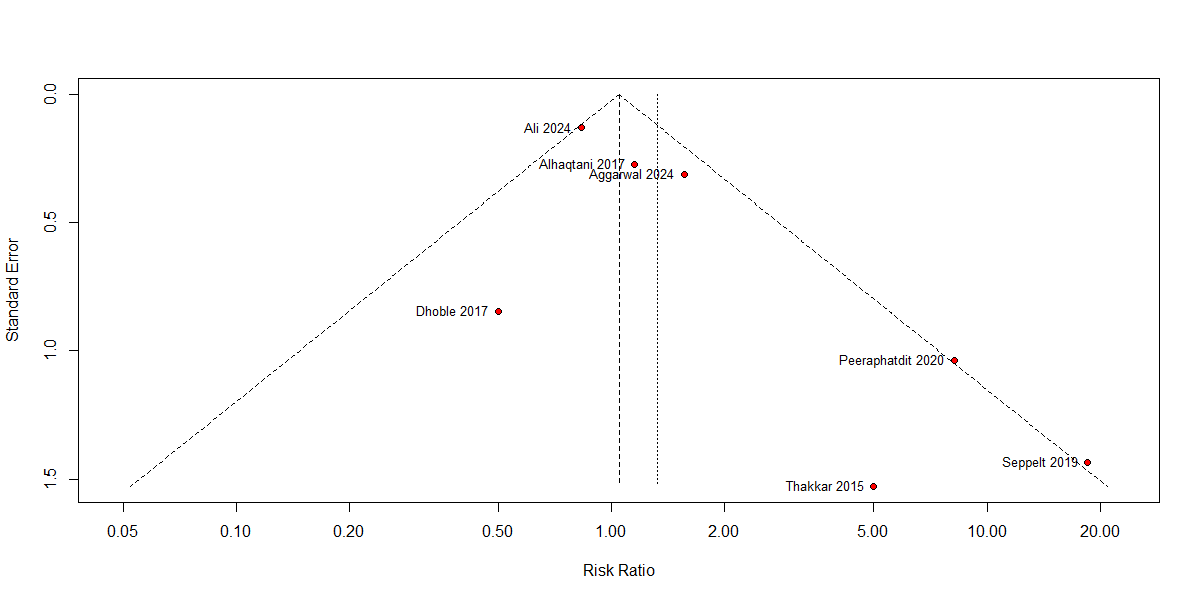


**Figure F6:** Funnel Plot for Myocardial Infarction


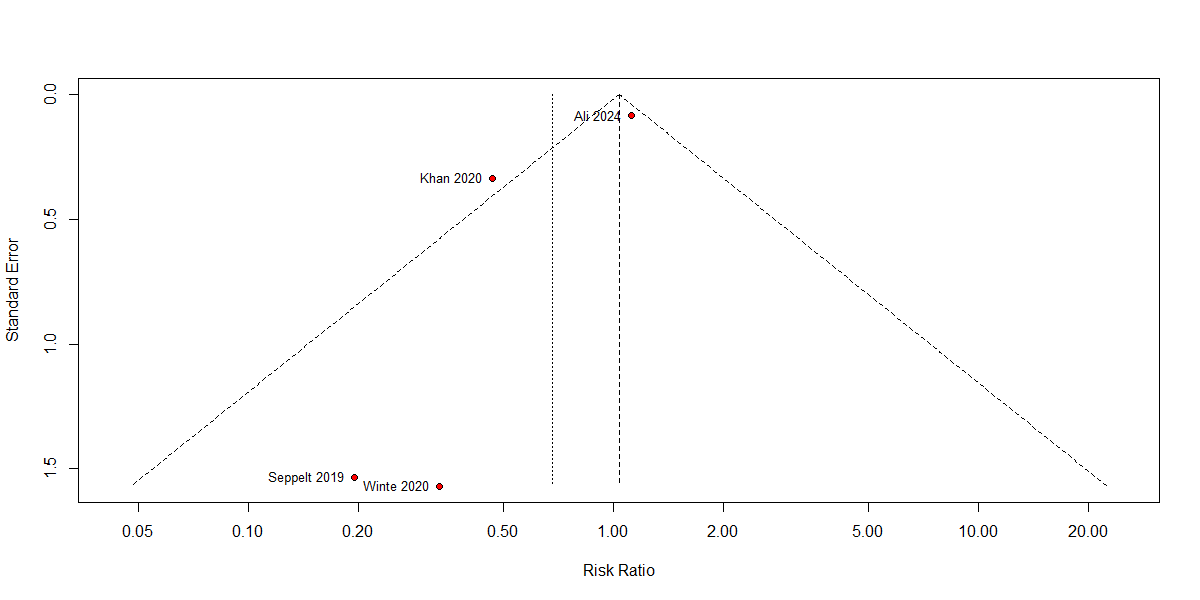


**Figure F7:** Funnel Plot for Hospital Stay
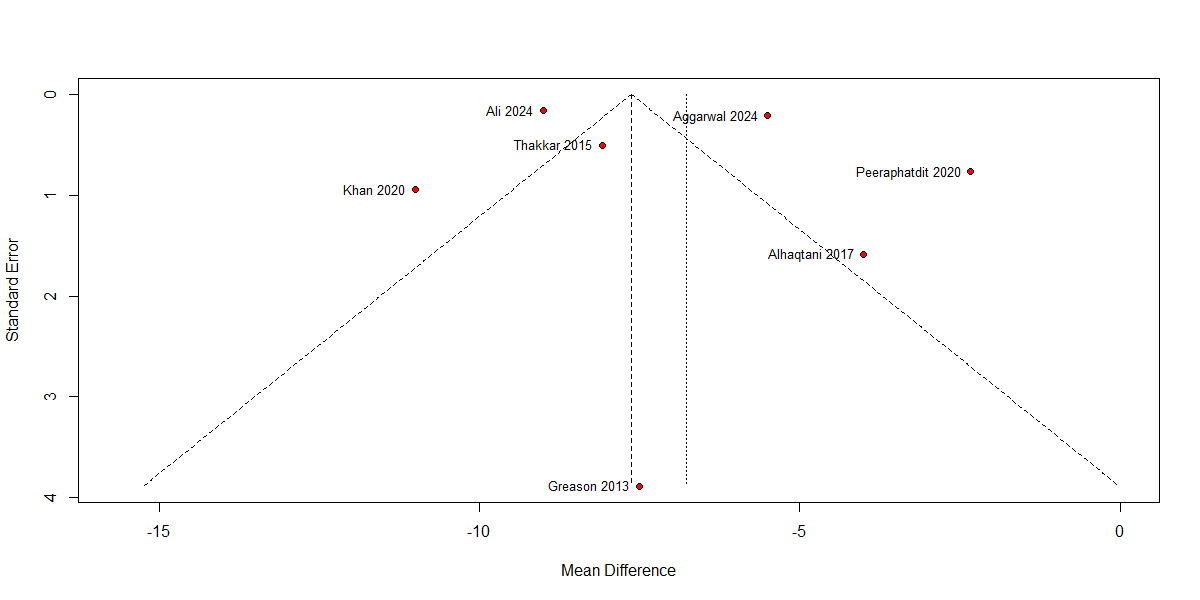


**Figure F8:** Funnel Plot for Hospital Mortality
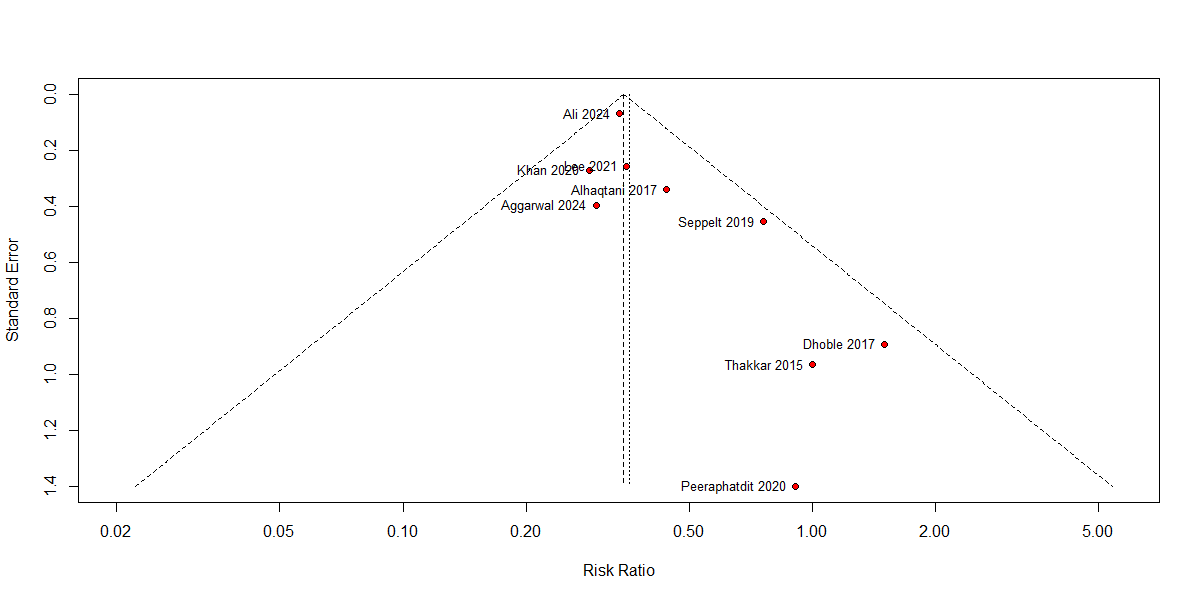


**Figure F9:** Funnel Plot for Hospital Charges


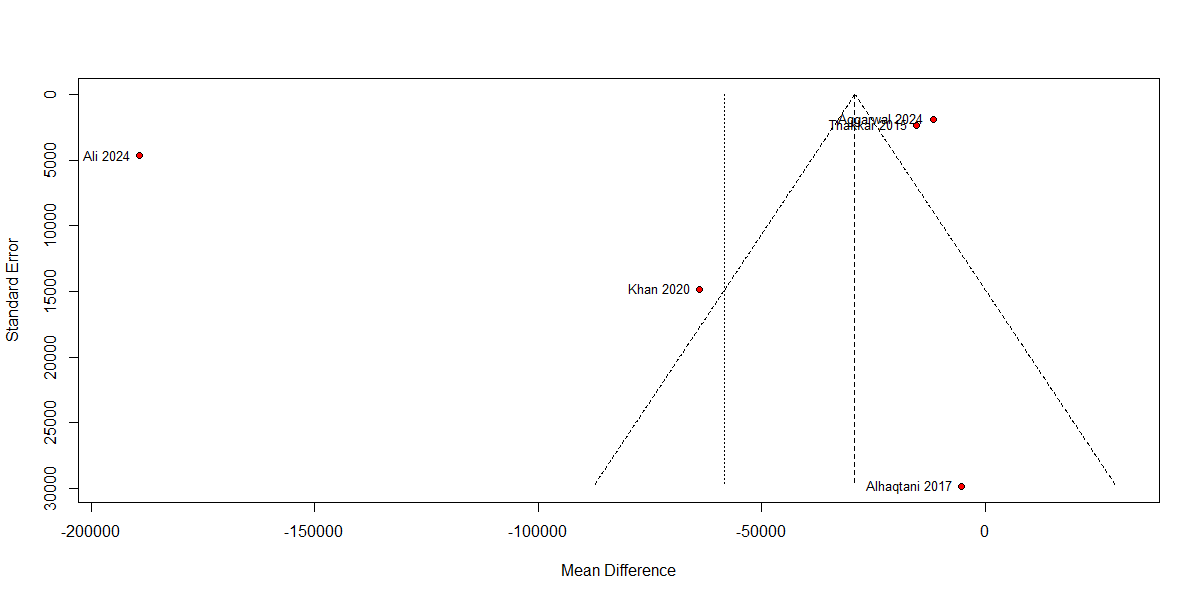


**Figure F10:** Funnel Plot for Cardiogenic Shock


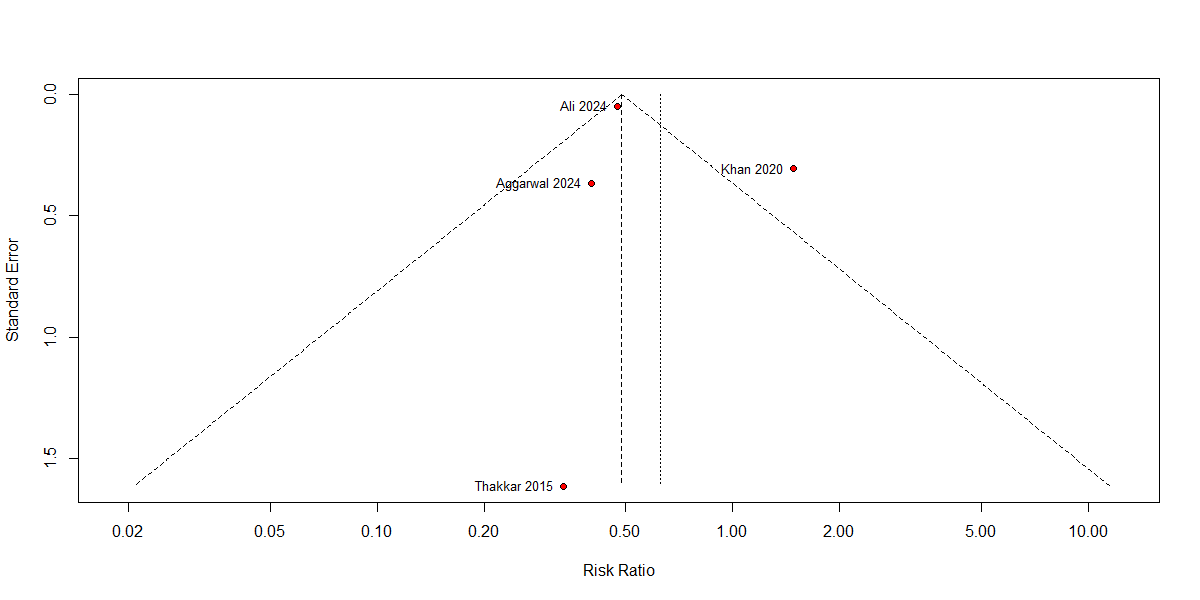


**Figure F11:** Funnel Plot for Cardiac Tamponade
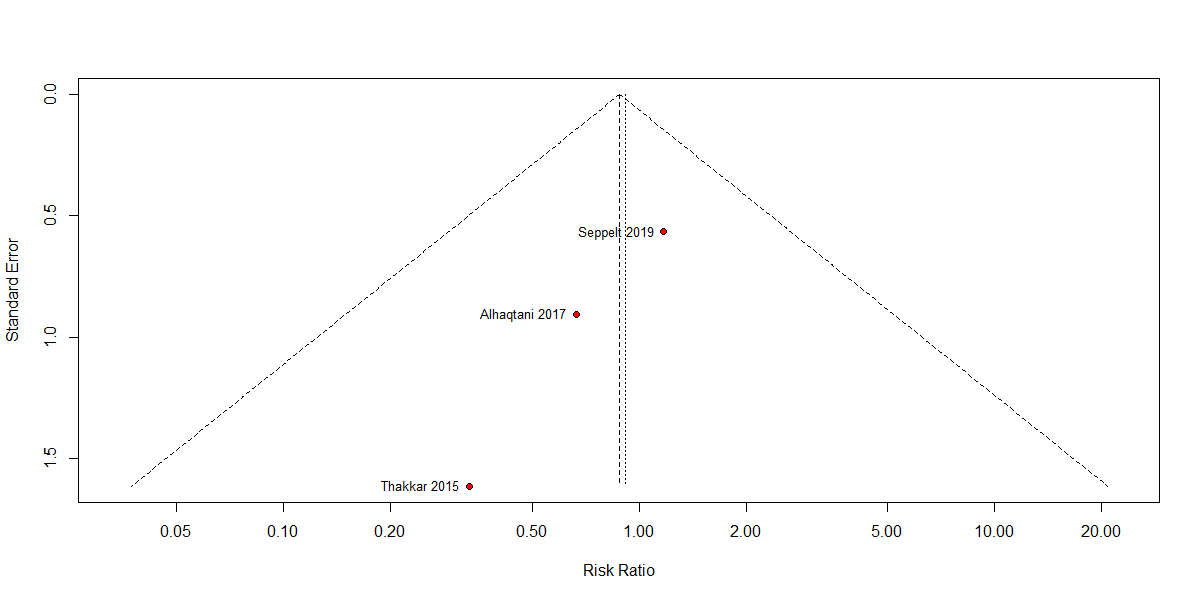


**Figure F12:** Funnel Plot for Cardiac Arrest


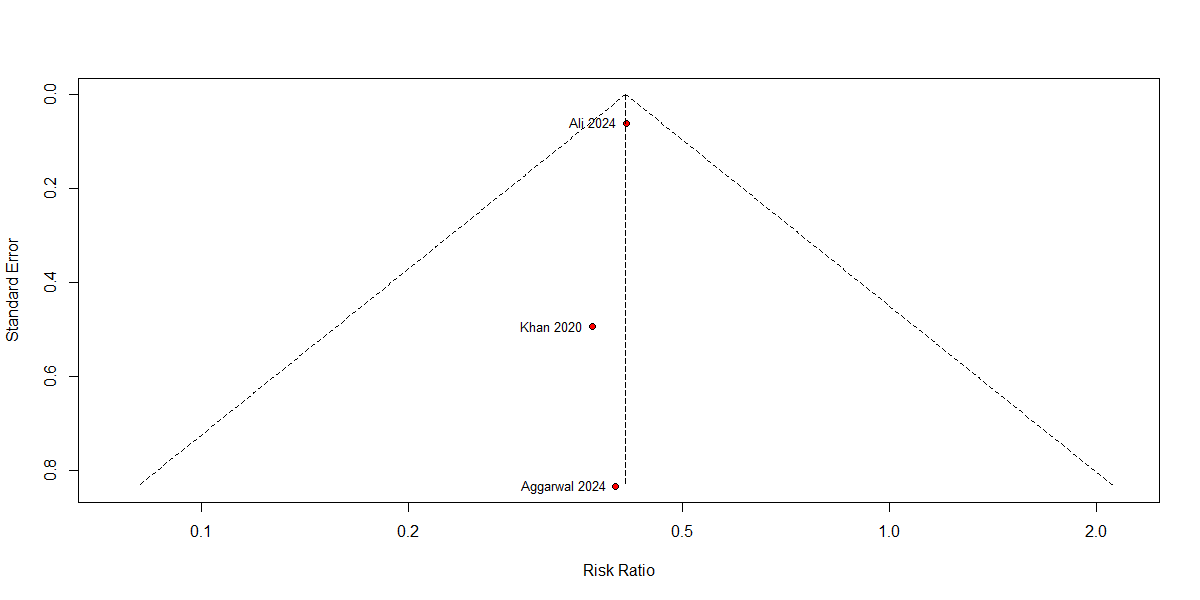


**Figure F13:** Funnel Plot for Bleeding


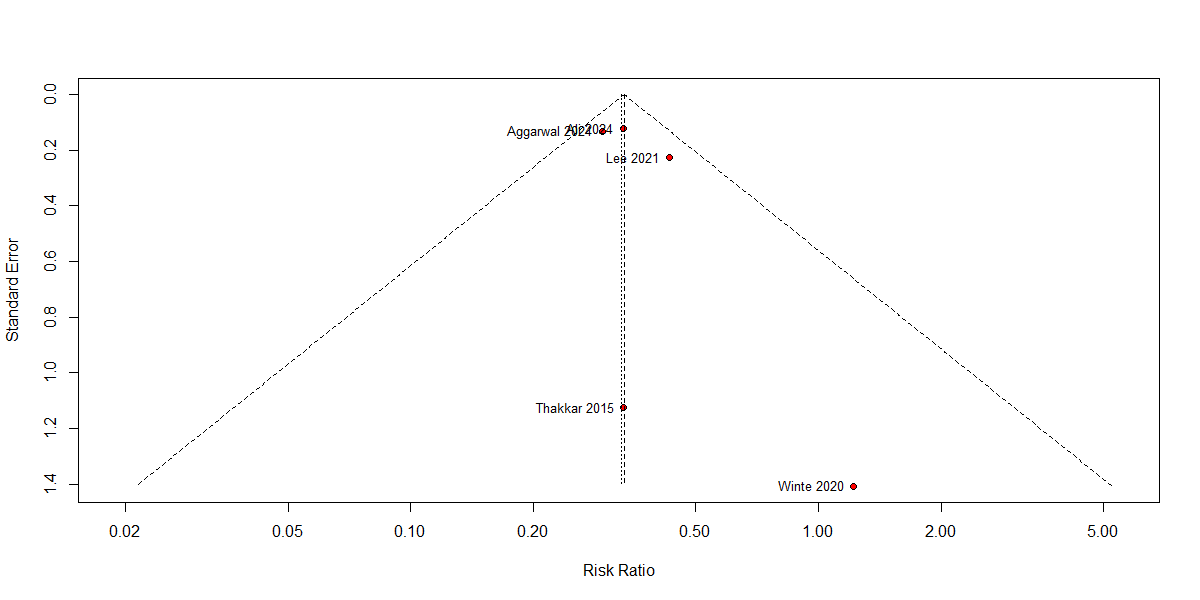


**Figure F14:** Funnel Plot for Blood Transfusion
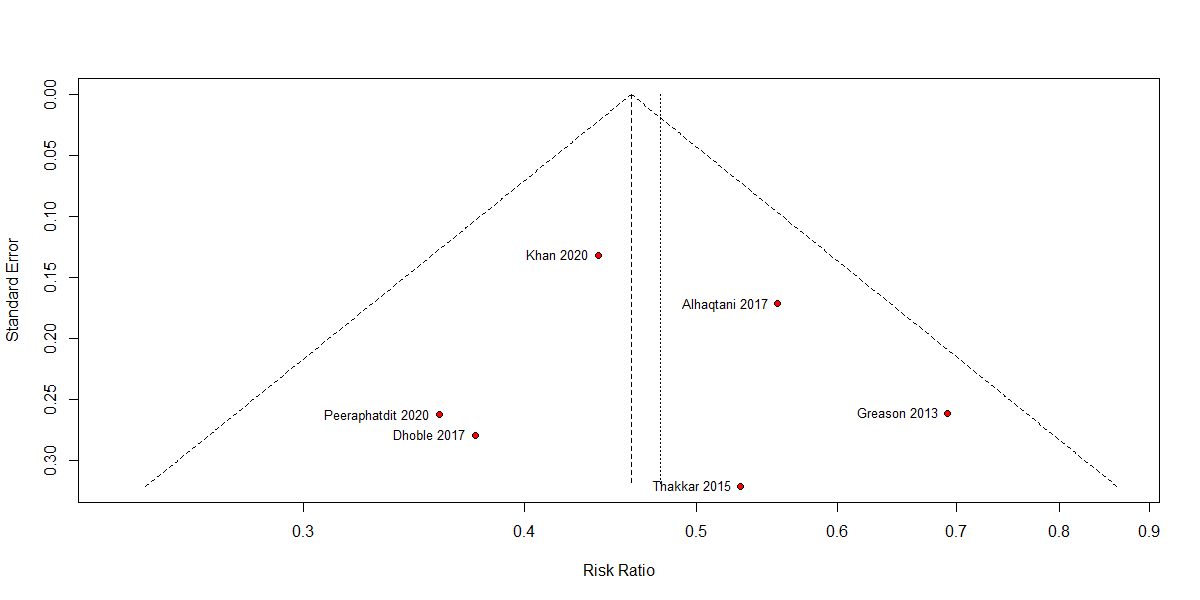


**Figure F14:** Funnel Plot for Acute Kidney Injury


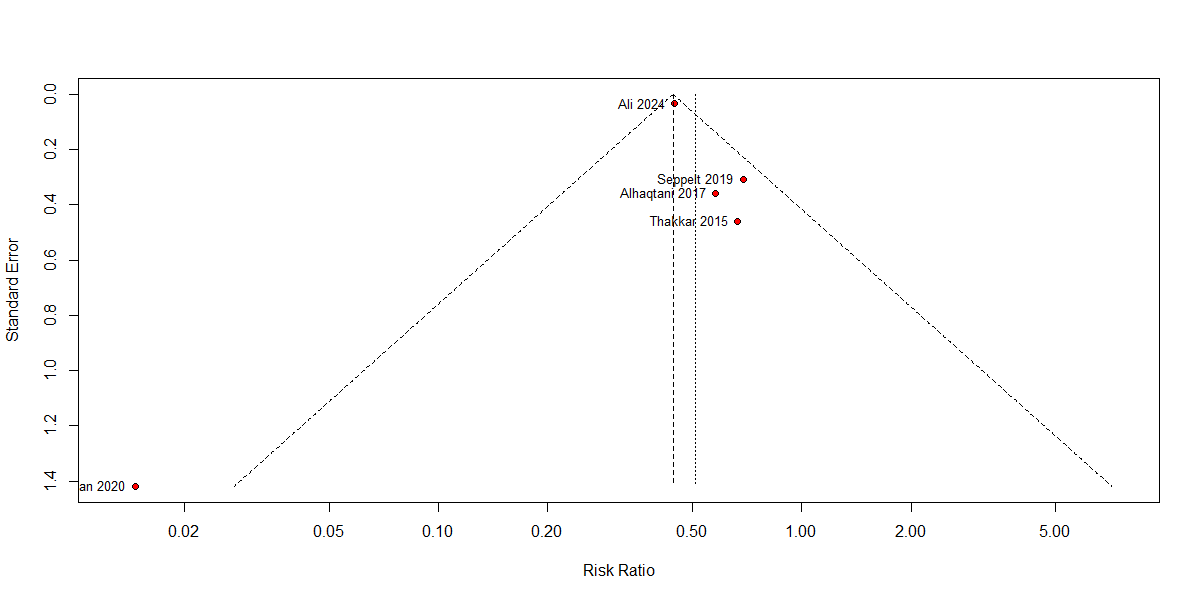


**Additional outcomes:**

**Figure S1** Forest Plot of Vascular Complications

**
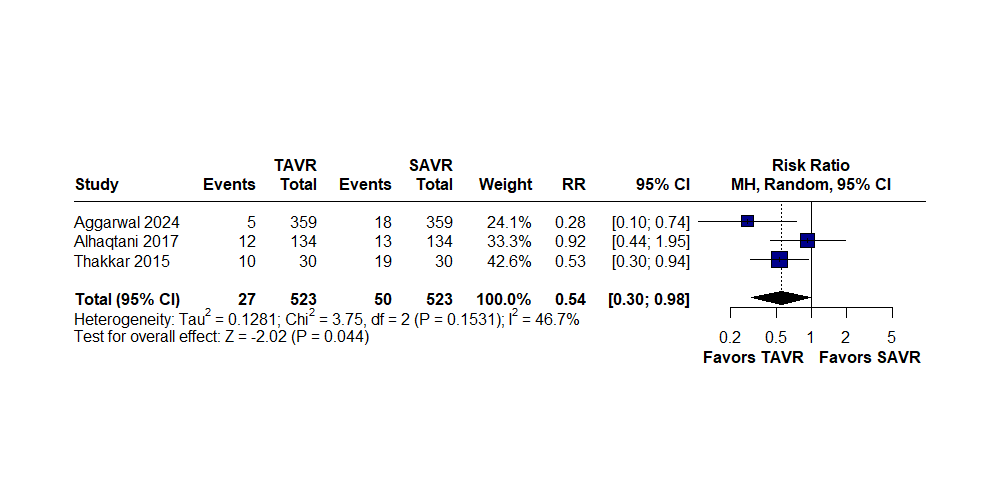
**

**Figure S2** Forest plot of length of hospital stay


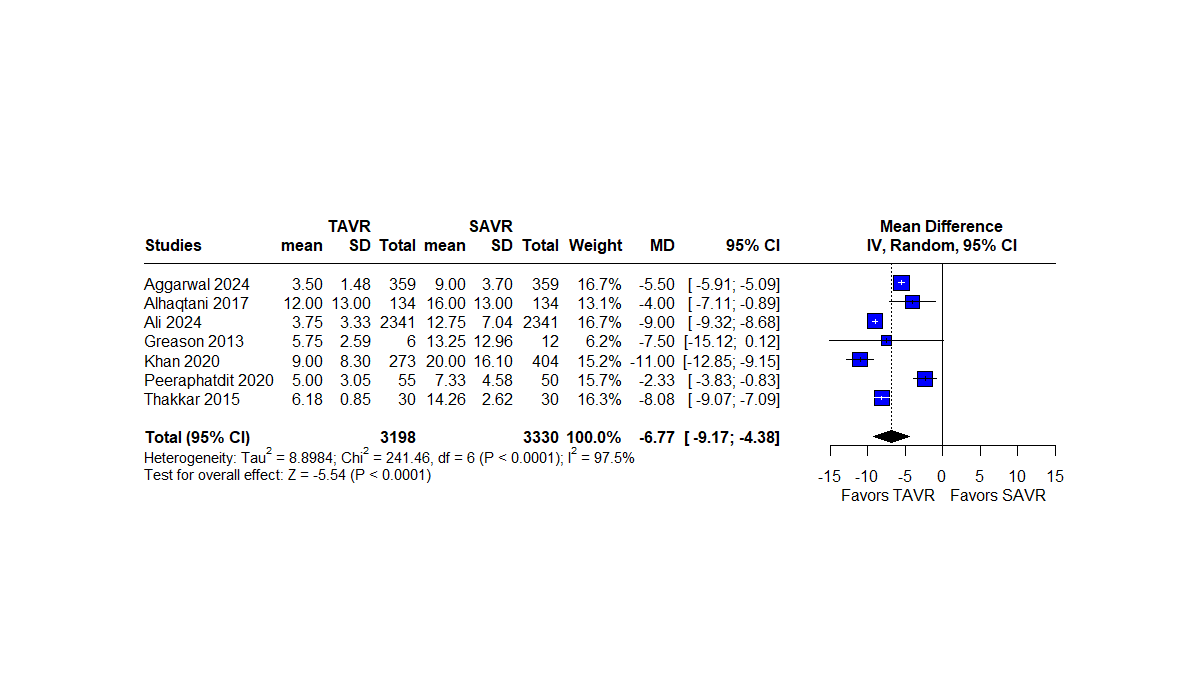


**Figure S3** Forest Plot of number of pacemaker implantations


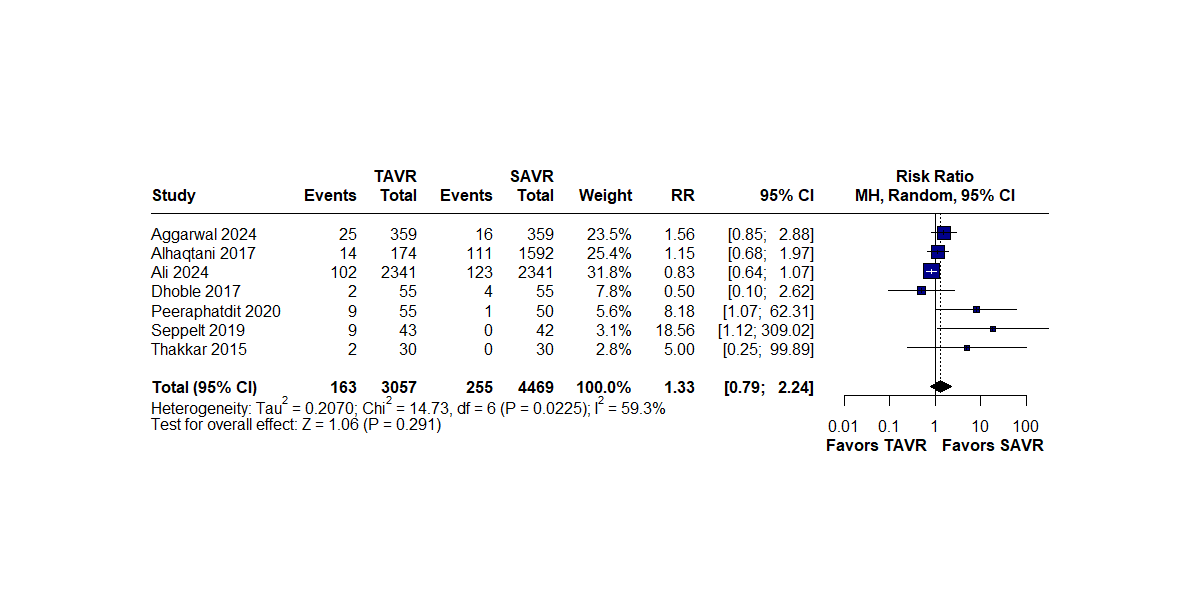


**Figure S4** Forest Plot of Vascular Complications requiring surgery


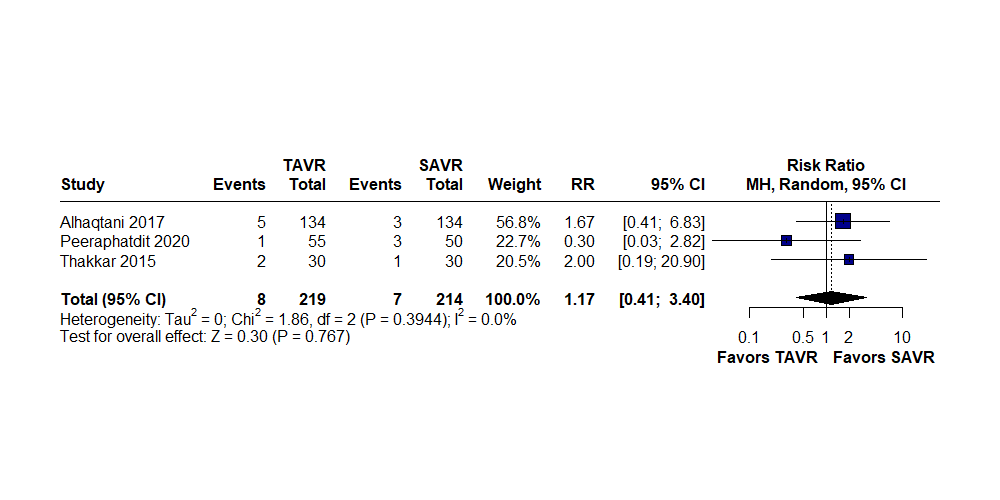


**Figure S5** Forest Plot of Myocardial Infarction


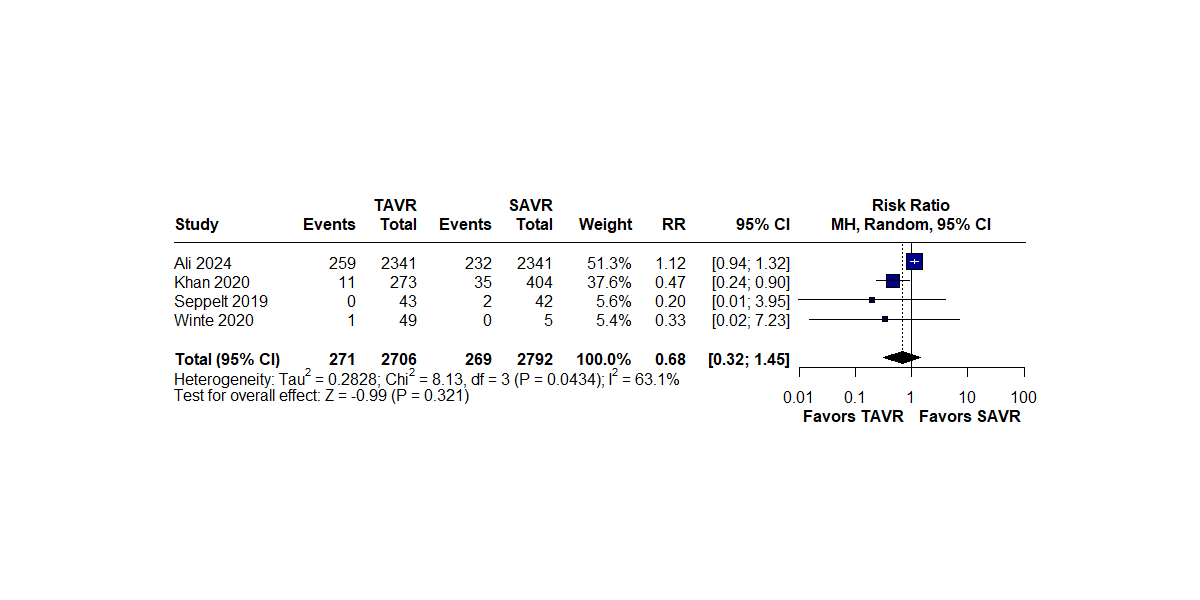


**Figure S6** Forest Plot of hospital charges


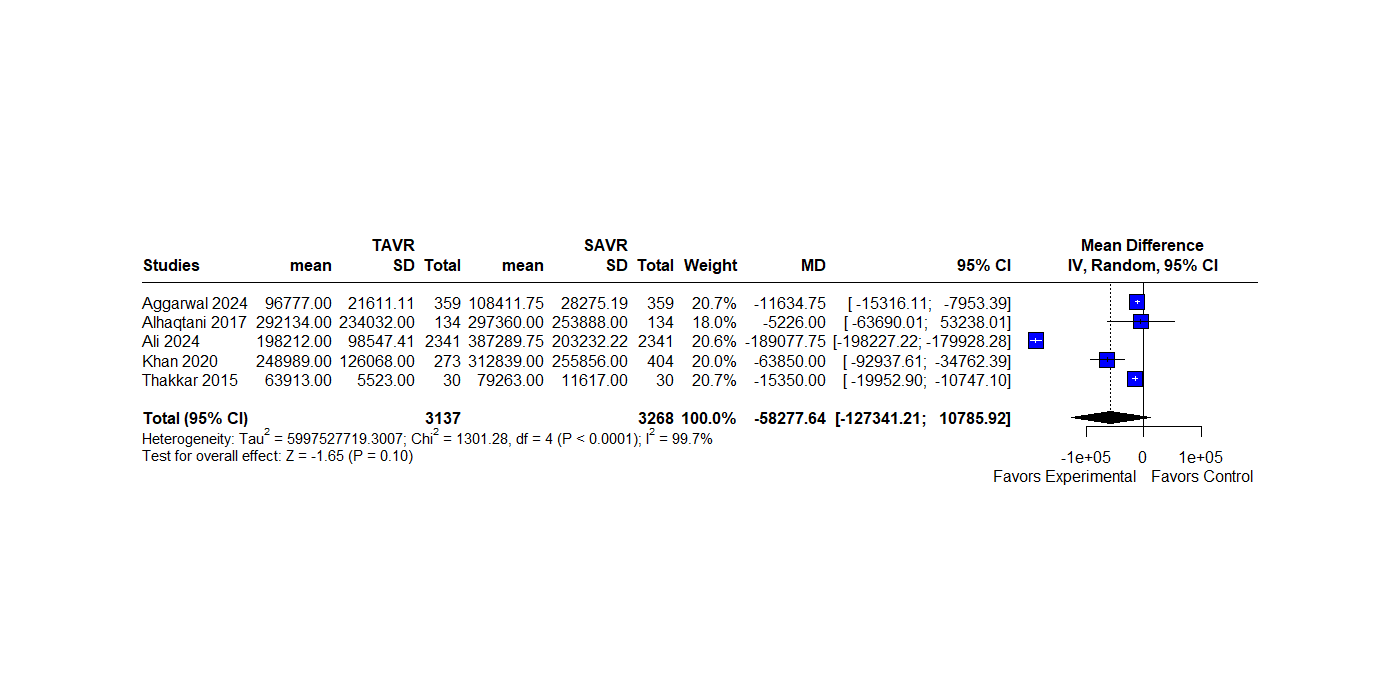


**Figure S7** Forest Plot of Cardiogenic Shock


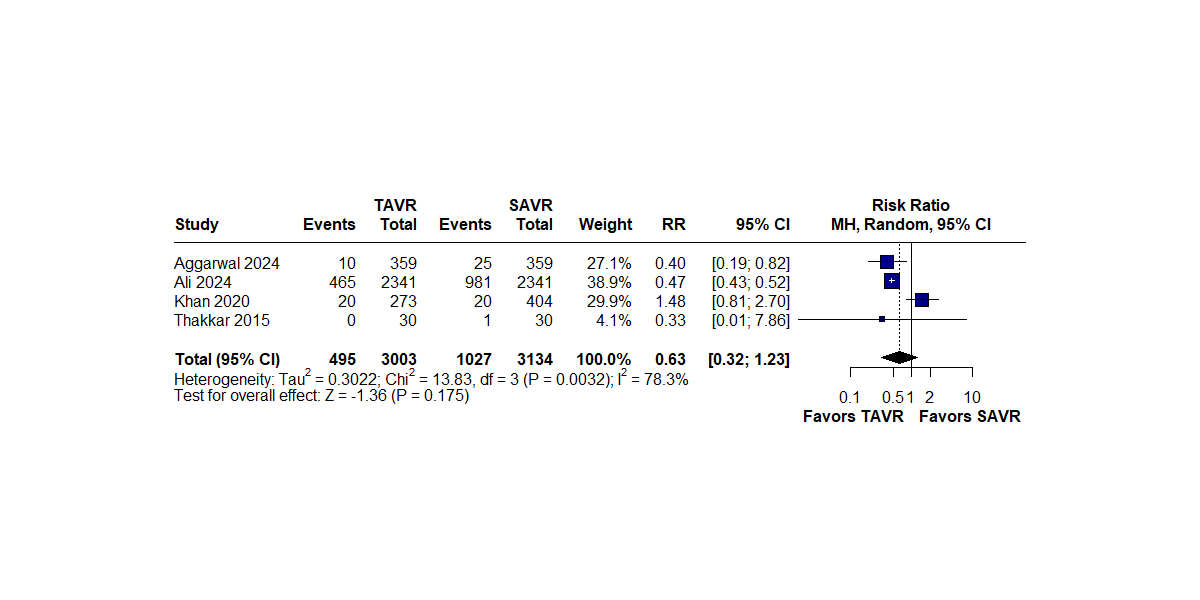


**Figure S8** Forest Plot of Cardiac Tamponade


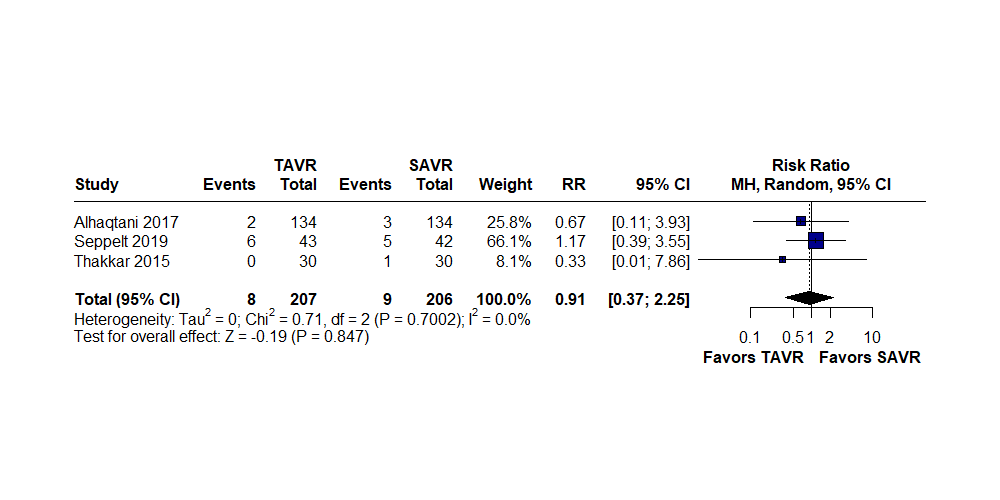


**Sensitivity:**

**Fig S9** Leave one out Sensitivity analysis for Cardiogenic Shock


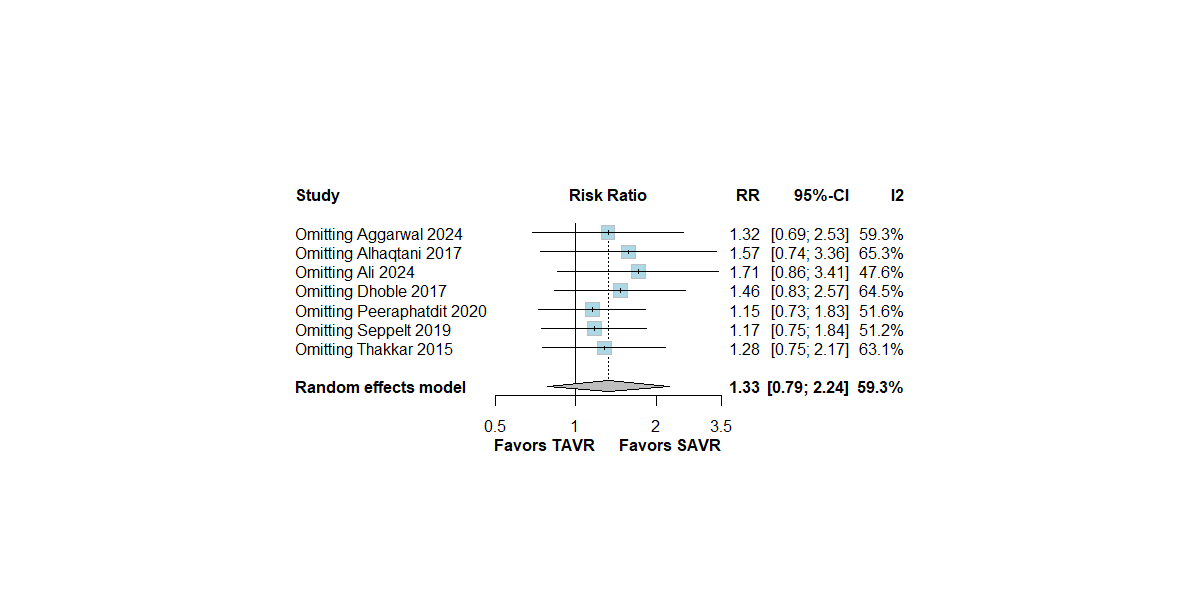


**Fig S10** Leave one out Sensitivity analysis for Myocardial Infarction


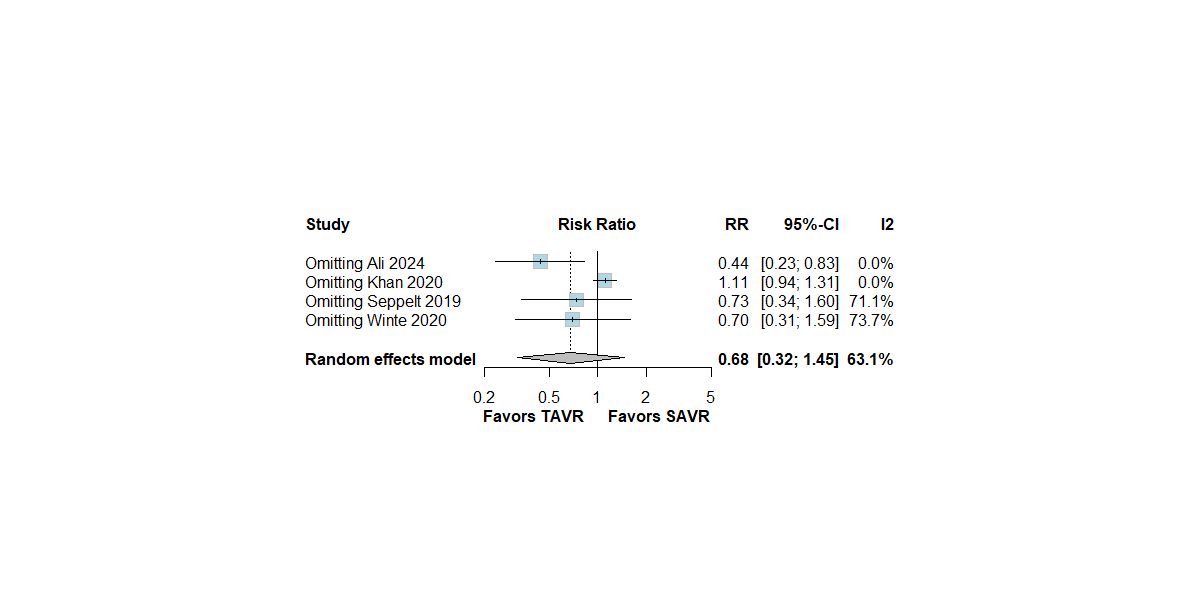


**Fig S11** Leave one out Sensitivity analysis for the number of pacemaker implantations


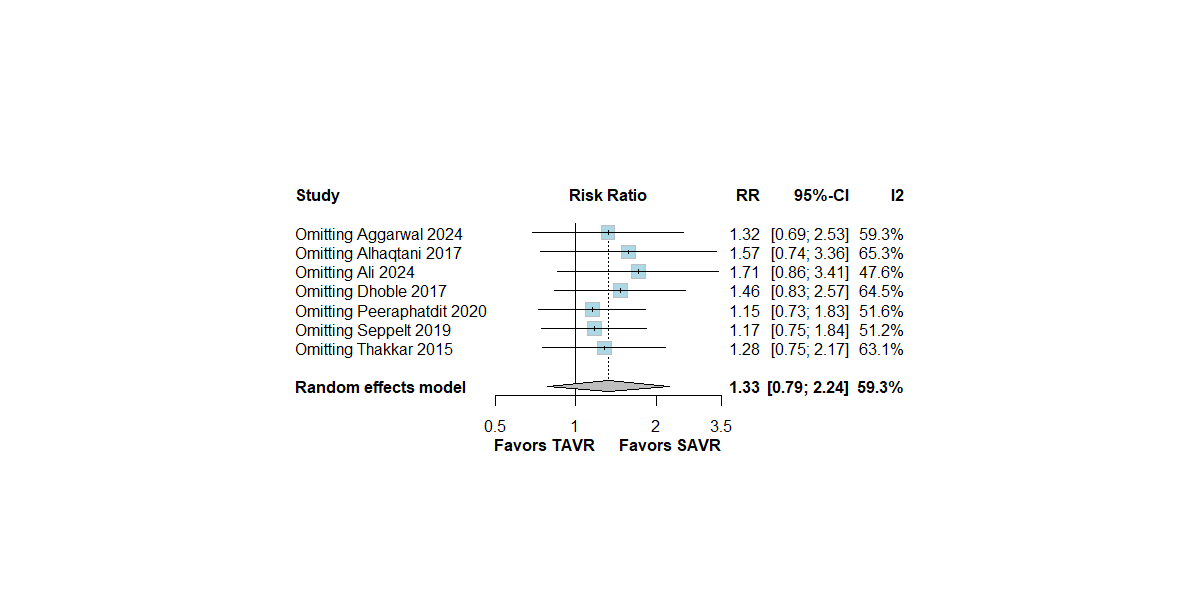


**Fig S12** Leave-one-out Sensitivity analysis for Acute Kidney Injury.


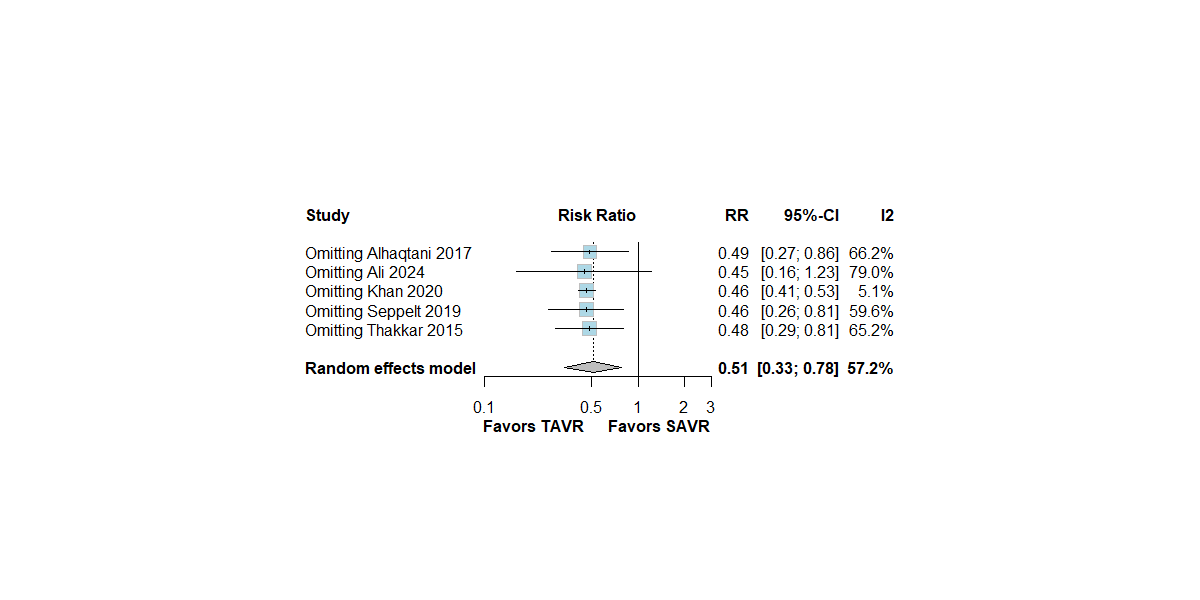
**PRISMA checklist**

| **Section and Topic** | **Item #** | **Checklist item** | **Location where item is reported** |  |  |
| --- | --- | --- | --- | --- | --- |
| **TITLE** | | |  |  |  |
| **Title** | **1** | **Identify the report as a systematic review.** | Page 1 |  |  |
| **ABSTRACT** | | |  |  |  |
| **Abstract** | **2** | **See the PRISMA 2020 for Abstracts checklist.** | Page 2 |  |  |
| **INTRODUCTION** | | |  |  |  |
| **Rationale** | **3** | **Describe the rationale for the review in the context of existing knowledge.** | Page 4 |  |  |
| **Objectives** | **4** | **Provide an explicit statement of the objective(s) or question(s) the review addresses.** | Page 4 |  |  |
| **METHODS** | | |  |  |  |
| **Eligibility criteria** | **5** | **Specify the inclusion and exclusion criteria for the review and how studies were grouped for the syntheses.** | Page 5 First paragraph |  |  |
| **Information sources** | **6** | **Specify all databases, registers, websites, organisations, reference lists and other sources searched or consulted to identify studies. Specify the date when each source was last searched or consulted.** | Page 4 |  |  |
| **Search strategy** | **7** | **Present the full search strategies for all databases, registers and websites, including any filters and limits used.** | Supplementary File table 1 |  |  |
| **Selection process** | **8** | **Specify the methods used to decide whether a study met the inclusion criteria of the review, including how many reviewers screened each record and each report retrieved, whether they worked independently, and if applicable, details of automation tools used in the process.** | Page 5 First Paragraph |  |  |
| **Data collection process** | **9** | **Specify the methods used to collect data from reports, including how many reviewers collected data from each report, whether they worked independently, any processes for obtaining or confirming data from study investigators, and if applicable, details of automation tools used in the process.** | Page 5 |  |  |
| **Data items** | **10a** | **List and define all outcomes for which data were sought. Specify whether all results that were compatible with each outcome domain in each study were sought (e.g. for all measures, time points, analyses), and if not, the methods used to decide which results to collect.** | Page 5 |  |  |
|  | **10b** | **List and define all other variables for which data were sought (e.g. participant and intervention characteristics, funding sources). Describe any assumptions made about any missing or unclear information.** | N/A |  |  |
| **Study risk of bias assessment** | **11** | **Specify the methods used to assess risk of bias in the included studies, including details of the tool(s) used, how many reviewers assessed each study and whether they worked independently, and if applicable, details of automation tools used in the process.** | Page 6 |  |  |
| **Effect measures** | **12** | **Specify for each outcome the effect measure(s) (e.g. risk ratio, mean difference) used in the synthesis or presentation of results.** | Page 6 |  |  |
| **Synthesis methods** | **13a** | **Describe the processes used to decide which studies were eligible for each synthesis (e.g. tabulating the study intervention characteristics and comparing against the planned groups for each synthesis (item #5)).** | Page 7 table 1 |  |  |
|  | **13b** | **Describe any methods required to prepare the data for presentation or synthesis, such as handling of missing summary statistics, or data conversions.** | N/A |  |  |
|  | **13c** | **Describe any methods used to tabulate or visually display results of individual studies and syntheses.** | N/A |  |  |
|  | **13d** | **Describe any methods used to synthesize results and provide a rationale for the choice(s). If meta-analysis was performed, describe the model(s), method(s) to identify the presence and extent of statistical heterogeneity, and software package(s) used.** | Page 7 |  |  |
|  | **13e** | **Describe any methods used to explore possible causes of heterogeneity among study results (e.g. subgroup analysis, meta-regression).** | Page 7 |  |  |
|  | **13f** | **Describe any sensitivity analyses conducted to assess robustness of the synthesized results.** | Page 7 |  |  |
| **Reporting bias assessment** | **14** | **Describe any methods used to assess risk of bias due to missing results in a synthesis (arising from reporting biases).** | Page 6 |  |  |
| **Certainty assessment** | **15** | **Describe any methods used to assess certainty (or confidence) in the body of evidence for an outcome.** | N/A |  |  |
| **RESULTS** | | |  |  |  |
| **Study selection** | **16a** | **Describe the results of the search and selection process, from the number of records identified in the search to the number of studies included in the review, ideally using a flow diagram.** | Figure 1 |  |  |
|  | **16b** | **Cite studies that might appear to meet the inclusion criteria, but which were excluded, and explain why they were excluded.** | Figure 1 |  |  |
| **Study characteristics** | **17** | **Cite each included study and present its characteristics.** | Table 1 |  |  |
| **Risk of bias in studies** | **18** | **Present assessments of risk of bias for each included study.** | Page 5 and supplementary table 3 |  |  |
| **Results of individual studies** | **19** | **For all outcomes, present, for each study: (a) summary statistics for each group (where appropriate) and (b) an effect estimate and its precision (e.g. confidence/credible interval), ideally using structured tables or plots.** | S1 to S8 page 13-16 supplementary file |  |  |
| **Results of syntheses** | **20a** | **For each synthesis, briefly summarise the characteristics and risk of bias among contributing studies.** | Supplementary file S3 |  |  |
|  | **20b** | **Present results of all statistical syntheses conducted. If meta-analysis was done, present for each the summary estimate and its precision (e.g. confidence/credible interval) and measures of statistical heterogeneity. If comparing groups, describe the direction of the effect.** | Page 8,9,10 |  |  |
|  | **20c** | **Present results of all investigations of possible causes of heterogeneity among study results.** | Page8, 9,10 |  |  |
|  | **20d** | **Present results of all sensitivity analyses conducted to assess the robustness of the synthesized results.** | Page 10 |  |  |
| **Reporting biases** | **21** | **Present assessments of risk of bias due to missing results (arising from reporting biases) for each synthesis assessed.** | N/A |  |  |
| **Certainty of evidence** | **22** | **Present assessments of certainty (or confidence) in the body of evidence for each outcome assessed.** | N/A |  |  |
| **DISCUSSION** | | |  |  |  |
| **Discussion** | **23a** | **Provide a general interpretation of the results in the context of other evidence.** | Page 10 |  |  |
|  | **23b** | **Discuss any limitations of the evidence included in the review.** | Page 11,12 |  |  |
|  | **23c** | **Discuss any limitations of the review processes used.** | Page 11,12 |  |  |
|  | **23d** | **Discuss implications of the results for practice, policy, and future research.** | Page 13 |  |  |
| **OTHER INFORMATION** | | |  |  |  |
| **Registration and protocol** | **24a** | **Provide registration information for the review, including register name and registration number, or state that the review was not registered.** | Methods page 4 |  |  |
|  | **24b** | **Indicate where the review protocol can be accessed, or state that a protocol was not prepared.** | N/A |  |  |
|  | **24c** | **Describe and explain any amendments to information provided at registration or in the protocol.** | N/A |  |  |
| **Support** | **25** | **Describe sources of financial or non-financial support for the review, and the role of the funders or sponsors in the review.** | Page 20 |  |  |
| **Competing interests** | **26** | **Declare any competing interests of review authors.** | Page 20 |  |  |
| **Availability of data, code and other materials** | **27** | **Report which of the following are publicly available and where they can be found: template data collection forms; data extracted from included studies; data used for all analyses; analytic code; any other materials used in the review.** | References |  |  |
